# ADSCs-Exo Attenuate HIRI in Miniature Pigs: Pharmacological Evidence for a Potential Association Between Copper-Associated Alterations and Ferroptosis-Related Responses

**DOI:** 10.3390/antiox15101335

**Published:** 2026-10-16

**Authors:** Xiangyu Lu, Pujun Li, Lei Cao, Tao Liu, Yajun Ma, Jiantao Zhang, Hongbin Wang

**Affiliations:** 1 College of Veterinary Medicine, Northeast Agricultural University, Harbin 150030, China; b220601009@neau.edu.cn (X.L.); pujunli@neau.edu.cn (P.L.); caolei@neau.edu.cn (L.C.); liutaotiger2015@neau.edu.cn (T.L.); b210601014@neau.edu.cn (Y.M.); zhangjiantao@neau.edu.cn (J.Z.); 2 College of Animal Science and Veterinary Medicine, Tianjin Agricultural University, Tianjin 300384, China; 3 Heilongjiang Provincial Key Laboratory of Pathogenic Mechanism for Animal Disease and Comparative Medicine, Harbin 150030, China

**Keywords:** copper homeostasis, cuproptosis-related markers, ferroptosis, ADSCs-Exo, HIRI

## Abstract

Hepatic ischemia–reperfusion injury (HIRI) involves multiple forms of regulated cell death, but the relationship between copper-associated alterations and ferroptosis-related responses remains incompletely understood. This study investigated whether pharmacological modulation of intracellular copper homeostasis was accompanied by corresponding changes in ferroptosis-associated responses, and vice versa, during HIRI, and evaluated whether adipose-derived mesenchymal stem cell-derived exosomes (ADSCs-Exo) attenuate injury-associated alterations under OGD/R and HIRI conditions. Primary piglet hepatocytes were subjected to oxygen–glucose deprivation/reoxygenation (OGD/R), followed by bidirectional pharmacological modulation of copper homeostasis and ferroptosis-associated responses. The major response patterns were further evaluated in a laparoscopic miniature pig model of HIRI combined with partial hepatectomy. OGD/R induced copper and iron dyshomeostasis, mitochondrial dysfunction, redox imbalance, alterations in cuproptosis-related markers, and ferroptosis-associated changes. Relative to the OGD/R group, ADSC-Exo treatment attenuated these injury-associated abnormalities. Pharmacological reduction in intracellular copper loading was accompanied by decreased Fe^2+^ accumulation and restoration of GSH, AIFM2, SLC7A11, and GPX4, whereas increased copper loading produced the opposite pattern. Conversely, pharmacological enhancement in ferroptosis-associated responses increased Cu^2+^ accumulation and aggravated alterations in the cuproptosis-related markers SLC31A1, FDX1, LIAS, and DLAT, whereas pharmacological suppression of ferroptosis-associated responses attenuated these changes. These bidirectional pharmacological response patterns are consistent with a potential reciprocal association between copper-associated alterations and ferroptosis-related responses but do not establish bona fide cuproptosis, direct molecular interaction, or target-specific causality. In miniature pigs, HIRI was accompanied by hepatic injury, mitochondrial dysfunction, hepatic copper accumulation, alterations in cuproptosis-related markers, ferroptosis-associated changes, and reduced GPX4 expression. Relative to untreated IRI animals, ADSC-Exo administration was associated with attenuation of these injury-related abnormalities. The coordinated changes in GPX4, TAX1BP1, and MAP1LC3B are hypothesis-generating and support the working hypothesis that TAX1BP1/MAP1LC3B-associated autophagic processes may contribute to GPX4 homeostasis; however, autophagic flux, GPX4 turnover, and direct TAX1BP1–GPX4 interaction were not assessed. Because a non-injured ADSC-Exo-only control was not included, the present study does not determine the independent effects of ADSCs-Exo under baseline conditions. Thus, the observed ADSC-Exo-associated effects should be interpreted specifically within the OGD/R and HIRI settings examined here.

## 1. Introduction

Hepatic ischemia–reperfusion injury (HIRI) is a major complication associated with liver transplantation and hepatic resection and is characterized by oxidative stress, mitochondrial dysfunction, inflammation, and multiple forms of regulated cell death [1,2,3,4,5]. Ferroptosis, an iron-dependent form of regulated cell death driven by iron accumulation and lipid peroxidation [6,7], has been recognized as an important contributor to HIRI, including steatotic liver models [8,9,10]. Cuproptosis is a recently identified copper-dependent form of regulated cell death characterized by the targeting of lipoylated tricarboxylic acid cycle proteins and subsequent mitochondrial proteotoxic stress [11,12]. Emerging evidence suggests that copper and iron metabolism are closely interconnected, and copper dysregulation can influence ferroptosis through multiple mechanisms [13,14,15,16,17]. In particular, mitochondrial dysfunction, glutathione (GSH) depletion, and GPX4 destabilization may represent potential molecular interfaces between copper dysregulation and ferroptosis-related injury [14,16,18,19,20,21,22]. However, whether copper-associated alterations are accompanied by reciprocal changes in ferroptosis-related responses during HIRI remains incompletely understood.

Adipose-derived mesenchymal stem cell-derived exosomes (ADSCs-Exo) have emerged as a promising cell-free therapeutic strategy because of their low immunogenicity, biological stability, and capacity to deliver bioactive molecules [23,24,25,26,27]. Previous studies, including our own, have shown that ADSCs-Exo ameliorate HIRI through multiple mechanisms, including regulation of lipid metabolism, endoplasmic reticulum stress, mitochondrial dysfunction, inflammatory responses, and ferroptosis [28,29,30,31,32,33,34,35,36]. In particular, our previous work demonstrated that ADSCs-Exo attenuated ferroptosis-related injury in this experimental system. However, these studies primarily focused on individual downstream injury pathways or pathway-specific effects of ADSCs-Exo. Whether copper homeostasis and ferroptosis-related responses change in a coordinated manner during HIRI and whether ADSC-Exo treatment is associated with simultaneous attenuation of these injury-related alterations have not been fully characterized.

Accordingly, the present study investigated whether pharmacological manipulation of intracellular copper homeostasis was accompanied by corresponding changes in ferroptosis-related responses and, conversely, whether pharmacological modulation of ferroptosis-associated responses was accompanied by alterations in copper homeostasis and cuproptosis-related markers. TTM and Cu(II)-Elesclomol were used to pharmacologically decrease or increase intracellular copper availability, whereas RSL3 and PKUMDL-LC-101-D013 were used to modulate ferroptosis-associated responses. This bidirectional pharmacological strategy was designed to evaluate response patterns consistent with a potential association between copper-associated alterations and ferroptosis-related processes, rather than to establish direct molecular interaction, target-specific causality, or bona fide cuproptosis. We further evaluated whether ADSC-Exo treatment was associated with coordinated attenuation of these injury-related abnormalities, with mitochondrial dysfunction, redox imbalance, and GSH/GPX4 homeostasis examined as potential points of convergence. Major response patterns were subsequently evaluated in a laparoscopic miniature pig model of HIRI combined with partial hepatectomy to assess their biological relevance in vivo.

## 2. Materials and Methods

### 2.1. Animals

Eighteen healthy Bama miniature pigs (4–6 months old, weighing 20–25 kg) were included in this study. The same cohort of 18 animals was used for both ADSC isolation and the subsequent laparoscopic HIRI experiment; no separate donor cohort was used for ADSC collection. Inguinal adipose tissue was collected for ADSC isolation and ADSC-Exo preparation before the animals entered the HIRI experimental protocol. The 18 animals were subsequently allocated to the Sham, IRI, and Exo groups (*n* = 6 per group), as described in Section 2.7. The pigs had free access to water and food and underwent clinical and laboratory examinations before inclusion. All animal experiments were approved by the Experimental Animal Welfare and Ethics Committee of Northeast Agricultural University (approval No. NEAUEC20230311; approval date: 10 January 2023).

### 2.2. Cells

The cells used in in vitro experiments include primary hepatocytes obtained from liver perfusion of newborn piglets and adipose mesenchymal stem cells extracted from inguinal adipose tissue of miniature pigs, with the latter being used for exosome extraction.

### 2.3. Preparation of ADSCs-Exo

The acquisition and identification of ADSCs and ADSCs-Exo were performed as described in the aforementioned article [28]. ADSCs were isolated from inguinal adipose tissue obtained from animals belonging to the same cohort of 18 miniature pigs subsequently used in the HIRI experiment, rather than from a separate donor population. Briefly, inguinal adipose tissue was obtained from miniature pigs under aseptic conditions. Under sterile conditions, ADSCs were isolated from the adipose tissue following digestion with type I collagenase, centrifugation, and other procedures. The cells were identified after being subcultured for 3 to 5 passages in an incubator. Subsequently, ADSCs were cultured in serum-free medium for 36 to 48 h, after which ADSCs-Exo were obtained via ultrafiltration and ultra-high-speed centrifugation. Following identification, the ADSCs-Exo were stored frozen for subsequent use. The antibodies used for the identification of ADSCs and ADSCs-Exo are listed in Table 1.

### 2.4. Hepatocyte (HC) Isolation, Culture and Identification

Primary hepatocytes were obtained using the in situ two-step liver perfusion method reported by Seglen [37]. Hepatocytes were first cultured in medium A (high glucose DMEM (HyClone, Logan, UT, USA), 10% FBS, 2 nM L-glutamine, 100 U/mL penicillin, 125 U/L insulin, 0.2 μg/mL dexamethasone) for 4 h. Subsequently, the cells were washed with PBS and then cultured in medium B (which differs from medium A only in the absence of FBS). The cells were identified by light microscopy observation, glycogen staining, and immunofluorescence immunostaining for the HC-specific marker protein CK-18 (10830-1-AP, Proteintech, Wuhan, China).

### 2.5. OGD/R Model

An in vitro oxygen–glucose deprivation/reoxygenation (OGD/R) model was established in primary porcine hepatocytes with reference to our previously reported experimental protocol [30], an in vitro OGD/R model of HCs was established to simulate in vivo HIRI. HCs were cultured in D-PBS solution supplemented with 2 nM L-glutamine and 100 U/mL penicillin-streptomycin, and placed in an environment of 37 °C, 5% CO_2_, and 95% N_2_ to induce OGD. After 2 h, the medium was replaced with medium B, and the cells were further cultured for 6 h in a normoxic environment at 37 °C with 5% CO_2_.

Before the formal pharmacological intervention experiments, preliminary concentration– and time–response screening was performed to determine appropriate working conditions for TTM, Cu(II)-Elesclomol, RSL3, and PKUMDL-LC-101-D013. Primary hepatocytes under OGD/R conditions were treated with graded concentrations of each compound for 1, 6, or 12 h, and cell viability was evaluated using the CCK-8 assay. Based on these concentration– and time–response profiles, 50 μM TTM, 10 nM Cu(II)-Elesclomol, 0.5 μM RSL3, and 5 μM PKUMDL-LC-101-D013 were selected as the working concentrations for the subsequent experiments, with pharmacological interventions administered 1 h before hypoxia exposure (Figure S1). These preliminary screening experiments were performed to define empirical working concentrations and exposure conditions based on cell viability and were not intended to demonstrate molecular target engagement or compound specificity.

For the formal intervention experiments, cells in the Exo group, OGD/R+Cu^2+^- group, OGD/R+Cu^2+^+ group, GPX4^−^+OGD/R group, and GPX4^+^+OGD/R group were administered corresponding doses of different intervention substances (10 μg/mL ADSCs-Exo, 50 μM TTM, 10 nM Cu(II)-Elesclomol, 0.5 μM RSL3, and 5 μM PKUMDL-LC-101-D013) 1 h before hypoxia exposure. The Cu+Exo group was given an intervention mixture of 10 nM Cu(II)-Elesclomol and 10 μg/mL ADSCs-Exo 1 h before hypoxia. The RSL3+Exo group was administered an intervention mixture of 5 μM PKUMDL-LC-101-D013 and 10 μg/mL ADSCs-Exo 1 h before hypoxia.

### 2.6. Cell Viability

Cells were seeded in 96-well plates and cultured normally for 6 h to recover. Cell viability in each group was then measured according to the instructions of the CCK-8 kit (MA0225, Meilunbio, Dalian, China).

### 2.7. HIRI Model

The animal flow was as follows: The same cohort of eighteen Bama miniature pigs described in Section 2.1 first provided inguinal adipose tissue for ADSC isolation and ADSC-Exo preparation and subsequently entered the HIRI experiment. No additional donor animals were used. After completion of ADSC collection, the eighteen animals were assigned individual identification numbers and randomly allocated, using a random-number table, to the Sham, IRI, and Exo groups (*n* = 6 per group). All eighteen animals entered the assigned experimental procedures and completed the study. The adipose-derived mesenchymal stem cell secretome promotes hepatic regeneration in miniature pigs after liver ischaemia–reperfusion combined with partial resection [38]. Briefly, animals in the Sham group underwent laparoscopic exploration only, whereas those in the IRI and Exo groups underwent 60 min of right hemihepatic ischemia followed by resection of the left hepatic lobe. Immediately after surgery, the Sham and IRI groups received 5 mL sterile PBS via the portal vein, whereas the Exo group received 200 μg ADSCs-Exo suspended in 5 mL PBS. Blood and liver samples were collected before surgery and at 1, 3, and 7 days after surgery.

Because the surgical procedures differed among groups, the investigators performing the operations were not blinded to group allocation. However, samples were coded before laboratory analysis, and investigators performing biochemical assays, Western blot quantification, and immunofluorescence analysis were blinded to group identity until data acquisition was completed. All six animals per group completed the experimental protocol. The animals and biological samples collected in this experiment were used as part of a broader research program involving multiple parallel biochemical and molecular analyses beyond the scope of the present manuscript. Because the available blood and tissue samples had to be allocated across a large number of analytical endpoints, three animals per group were randomly selected before laboratory analysis for the set of biochemical and molecular measurements reported in in the in vivo vehicle experiment results and the corresponding supplementary analyses. The same three animals were used across these endpoints to maintain consistency among the analyses. Serum ALT and AST, although requiring only a small sample volume for individual measurement, were also analyzed within this same predefined analytical subset. Samples from the remaining animals were allocated to other parallel analyses within the broader research program and are no longer available for retrospective measurement of ALT and AST. Therefore, the effective analytical sample size for the endpoints reported in the in vivo vehicle experiment results was *n* = 3 independent animals per group.

### 2.8. RT-qPCR

Total RNA was extracted from liver tissues using the Trizol method, quantified, and reverse-transcribed into cDNA (CW2020M, CWBIO, Jiangsu, China). The cDNA was then labeled with SYBRI green fluorescent dye (CW3360M, CWBIO, Jiangsu, China), and target genes were amplified using the three-step method on a LightCycler 480 instrument (Roche, Basel, Switzerland). The relative mRNA expression levels of the target genes were calculated using the 2^−ΔΔCt^ method, with ACTB serving as the internal reference gene, as previously described by Livak and Schmittgen [39]. Prior to normalization, the raw Ct values of ACTB were re-examined across the experimental groups and sampling time points and showed no systematic group- or time-dependent variation, supporting its stability and suitability as the internal reference gene in the present dataset. Primers were synthesized by UW Genetics, and the primer sequences together with the corresponding NCBI Reference Sequence (RefSeq) accession numbers of the target genes are provided in Table 2.

### 2.9. Western Blotting

Total proteins were extracted from HCs and liver tissue samples by lysing them in RIPA lysis buffer (MA0151, Meilun Bio, Dalian, China) containing 1% protease inhibitor (P1005, Beyotime, Shanghai, China) and 1% phosphatase inhibitor (PR20015, Proteintech, Wuhan, China). The total protein concentration was determined using the BCA method, and an appropriate amount of normal saline and protein loading buffer (P0015, Beyotime, Shanghai, China) was added to adjust to a uniform concentration. Subsequently, electrophoresis was performed on SDS-PAGE gels with different concentrations, followed by transfer of proteins onto NC membranes (Cytiva, Marlborough, MA, USA) or PVDF membranes (Merck Millipore, Darmstadt, Germany) using the wet transfer method. After blocking in 1% PVP4000 solution for 20 min, the membranes were incubated with the corresponding primary antibodies overnight at 4 °C. On the following day, the membranes were washed with TBST and incubated with horseradish peroxidase-conjugated secondary antibodies for 1 h at room temperature. Finally, ECL luminescent solution (MA0186, Meilun, Dalian, China) was added dropwise to the bands, which were then developed using the Tanon 5200 system. ACTB or GAPDH was used as the loading control for Western blot normalization, as appropriate. Before densitometric quantification, the corresponding ACTB/GAPDH loading-control signals were reviewed across the experimental groups and showed no systematic variation, supporting their stability under the experimental conditions used in this study. The average optical density of each protein was calculated using ImageJ (Java 1.8.0_112 64-bit). The antibodies used for each target protein are listed in Table 3.

### 2.10. Liver Function Assay

According to the instructions of the kit, the cell culture supernatants of each group were collected, centrifuged at 400× *g* for 5 min, and the supernatants were harvested to determine the changes in the contents of alanine transaminase (ALT) and aspartate transaminase (AST) (Nanjing Jiancheng Bioengineering, Nanjing, China).

According to the manufacturer’s instructions, an appropriate amount of PBS was added to the cell pellets of each group, followed by ultrasonic disruption. The changes in the contents of triglycerides (TGs) and total cholesterol (T-CHOL) (Nanjing Jiancheng Bioengineering, Nanjing, China) were then determined.

Blood samples from miniature pigs in each group were centrifuged at 3500 rpm for 10 min. The levels of ALT, AST, TG, and T-CHOL in the upper serum were measured using a BioTek blood biochemical analyzer (TBA-2000FR, Canon Medical. Systems Corporation, Tochigi, Japan).

### 2.11. Cu^2+^ and Fe^2+^ Assay

According to the manufacturer’s instructions, Reagent 4 lysis buffer was added to the collected cell samples, which were thoroughly mixed and lysed on ice for 10 min. The lysates were centrifuged at 12,000× *g* for 10 min at 4 °C, and the supernatants were collected for determination of intracellular Cu^2+^ content (E-BC-K775-M, Elabscience, Wuhan, China). Liver tissue homogenates were prepared using double-distilled water, and the supernatants obtained after centrifugation were used to determine hepatic Cu^2+^ content (E-BC-K300-M, Elabscience, Wuhan, China). Intracellular and hepatic Cu^2+^ contents were expressed as μmol/g protein.

For intracellular Fe^2+^ measurement, Reagent 1 was added to the collected cells, followed by lysis on ice for 10 min and centrifugation at 15,000× *g* for 10 min. The supernatants were collected for Fe^2+^ determination (E-BC-K881-M, Elabscience, Wuhan, China), with the results expressed as nmol/10^6^ cells. A 10% liver tissue homogenate was prepared using physiological saline and centrifuged at 2500 rpm for 10 min, and the supernatants were collected for determination of hepatic Fe^2+^ content (A039-2-1, Nanjing Jiancheng Bioengineering Institute, Nanjing, China), expressed as μmol/g protein.

### 2.12. Lipid Peroxidation (LPO) Assay

After the cells were washed with PBS, an appropriate amount of BODIPY 581/591 C11 working solution was added, and the cells were incubated in a cell culture incubator for 20 min. Finally, observations were made and images were captured under a fluorescence microscope. When detecting the reduced products, the excitation wavelength was set to 581 nm and the emission wavelength to 591 nm; when detecting the oxidized products, the excitation wavelength was set to 488 nm and the emission wavelength to 510 nm.

Liver tissues were homogenized in PBS, and the supernatant was collected after centrifugation. The changes in the content of lipid peroxidation (LPO) in liver tissues of each group were determined according to the manufacturer’s instructions (E-BC-K176-M, Elabscience, Wuhan, China).

### 2.13. Measurement of ATP Content

Cells were collected and resuspended in PBS, followed by ultrasonic disruption. After centrifugation at 3000 rpm for 20 min at 4 °C, the supernatant was retained for later use. A 10% liver tissue homogenate was prepared with PBS, centrifuged at 5000× *g* for 10 min at 4 °C, and the supernatant was reserved for subsequent use. The changes in ATP content in cells and tissues were determined according to the manufacturer’s instructions (MM-3267501, mmbio, Yancheng, China).

### 2.14. Measurement of Mitochondrial Membrane Potential (MMP)

Cells were cultured in 6-well plates. At the end of the experiment, mitochondrial membrane potential in each group of cells was detected using a JC-1 staining kit (E-CK-A301, Elabscience, Wuhan, China). JC-1 staining solution was added to the cells and incubated in a cell culture incubator for 20 min. After washing with PBS, observations were made and images were captured under a fluorescence microscope. For the detection of JC-1 monomers, the excitation wavelength was set to 490 nm and the emission wavelength to 530 nm; for the detection of JC-1 aggregates, the excitation wavelength was set to 525 nm and the emission wavelength to 590 nm.

### 2.15. Pyruvate Dehydrogenase (PDH) Assay

According to the reagent manufacturer’s instructions, Reagent 1 was used as the homogenization medium to prepare cell and liver tissue homogenates. After centrifugation at 12,000× *g* for 10 min, the supernatants were collected to determine the changes in PDH content in cells and liver tissues of each group (E-BC-K650-M, Elabscience, Wuhan, China).

### 2.16. Mitochondrial Respiratory Chain Complex I Assay

Cells were collected, and according to the kit instructions, Reagent 1 and Reagent 2 were added, mixed well, followed by ultrasonic disruption. After centrifugation at 10,000× *g* for 3 min, the supernatants were collected to determine the changes in the content of mitochondrial respiratory chain complex I in each group of cells (E-BC-K834-M, Elabscience, Wuhan, China).

### 2.17. Measurement of GSH Content

Cells were collected, rinsed with PBS, and subjected to low-speed centrifugation. After centrifugation, 0.3 mL of Reagent 4 was added to the cells. Following ultrasonic disruption, the mixture was centrifuged at 3500 rpm for 10 min at 4 °C, and the supernatant was collected and stored for subsequent use. Liver tissue samples were weighed, and Reagent 4 was added at a weight-to-volume ratio of 1:4 (*w*/*v*) for tissue homogenization. The tissue homogenate was centrifuged at 3500 rpm for 10 min at 4 °C, and the supernatant was collected and stored for subsequent use. According to the manufacturer’s instructions of the kit, the content of reduced GSH in cells was determined. Meanwhile, the contents of GSH in liver tissue were measured.

### 2.18. Immunofluorescence Staining

Fresh liver tissues were fixed with paraformaldehyde, dehydrated, embedded, and then sectioned. The sections were dewaxed, subjected to antigen retrieval, washed with PBS, and then blocked with a solution containing 5% BSA. The culture medium of cell climbing slices was aspirated, and the cells were fixed with paraformaldehyde. After washing with PBS, PBS containing 0.1% Triton X-100 was added dropwise for permeabilization. Following another PBS wash, 5% BSA was added for blocking. After washing with PBS, the primary antibodies against MAP1LC3B (T55990, Abmart, Shanghai, China), GPX4 (67763-1-Ig, Proteintech, Wuhan, China), and TAX1BP1 (14424-1-AP, Proteintech, Wuhan, China) were mixed in proportion, added dropwise onto the sections or cell climbing slices, and incubated overnight at 4 °C in the dark. On the following day, after washing with PBS, three species-matched fluorescent secondary antibodies were added and incubated at room temperature in the dark for 30–60 min. After washing with PBS, DAPI staining solution was added dropwise to label cell nuclei. Following another PBS wash, the samples were mounted with anti-fluorescence quenching medium. Finally, observations were made and images were captured under a fluorescence microscope or laser scanning confocal microscope.

### 2.19. Statistical Analysis

Statistical analyses were performed using SPSS software (version 22.0; IBM Corp., Armonk, NY, USA). Data are expressed as the mean ± standard deviation (SD). For the in vitro experiments, *n* = 3 represents three independently performed experiments, unless otherwise indicated. When technical replicates were included within an independent experiment, their values were averaged before statistical analysis and were not treated as independent observations.

For the in vivo experiments, six animals were included in each experimental group. For the biochemical and molecular endpoints presented in the in vivo vehicle experiment results and the corresponding supplementary analyses, including serum ALT and AST, *n* = 3 represents three independent animals per group randomly selected from the six animals before the corresponding assays, as described in Section 2.7. These three animals constituted the analytical subset used consistently across the reported in vivo endpoints.

For comparisons among multiple independent groups, one-way analysis of variance (ANOVA) followed by Tukey’s multiple-comparison test was used. For the in vivo variables assessed before surgery and at 1, 3, and 7 days after surgery, statistical comparisons among the Sham, IRI, and Exo groups were performed separately at each sampling time point using one-way ANOVA followed by Tukey’s multiple-comparison test. Thus, the present analysis was designed to evaluate between-group differences at each predefined sampling time point rather than longitudinal changes over time, and no group × time interaction was tested.

Tukey-adjusted *p*-values were used for post hoc pairwise comparisons to account for multiple comparisons among groups within each endpoint and sampling time point. A *p*-value < 0.05 after multiple-comparison adjustment was considered statistically significant. No formal a priori power calculation was performed for the present study. Given that the in vivo biochemical and molecular endpoints reported in the in vivo vehicle experiment results were analyzed using three independent animals per group, these analyses should be regarded as exploratory and interpreted cautiously with respect to statistical precision and generalizability. Confirmation in larger, prospectively powered cohorts will be required.

## 3. Results

### 3.1. Identification of ADSCs and ADSCs-Exo

The characterization of ADSCs and ADSCs-Exo was performed as described in our previous study [29]. Briefly, the isolated ADSCs exhibited a typical spindle-shaped morphology and grew adherently in a characteristic whirlpool-like pattern. Flow cytometric analysis showed high expression of the mesenchymal stem cell-associated positive markers CD29, CD44, and CD90, whereas the negative marker CD11b was expressed at a low level. Following lineage-specific induction, ADSCs successfully differentiated into adipocytes, hepatocyte-like cells, and osteoblasts, confirming their multilineage differentiation potential.

The isolated ADSCs-Exo displayed the characteristic cup-shaped morphology with a bilayer membrane structure. They exhibited high expression of the exosomal positive markers CD63, CD81, and TSG101, while the negative marker GRP94 was expressed at a low level [35]. Particle size analysis further demonstrated that the diameter of ADSCs-Exo was predominantly within the typical exosomal size range of 30–150 nm. Collectively, these findings confirmed that the isolated ADSCs and ADSCs-Exo exhibited morphological characteristics, immunophenotypic profiles, and functional properties consistent with the established criteria for ADSCs and exosomes, respectively, with satisfactory purity for subsequent experiments.

### 3.2. Isolation and Characterization of Primary Hepatocytes

The isolation, culture, and characterization of primary hepatocytes from neonatal piglets used in this study have been systematically described in our previous work [30]. Briefly, the isolated hepatocytes exhibited good attachment and growth characteristics, with predominantly irregular polygonal morphology, tight intercellular connections, and the presence of mononucleated, binucleated, and multinucleated cells. Positive glycogen staining and CK-18 immunofluorescence further confirmed their typical hepatocyte characteristics. These findings verified that the isolated cells were primary hepatocytes of sufficient quality for subsequent experiments.

### 3.3. ADSC-Exo Pretreatment Attenuates Injury-Associated Changes Under OGD/R Conditions

**Figure 1:**
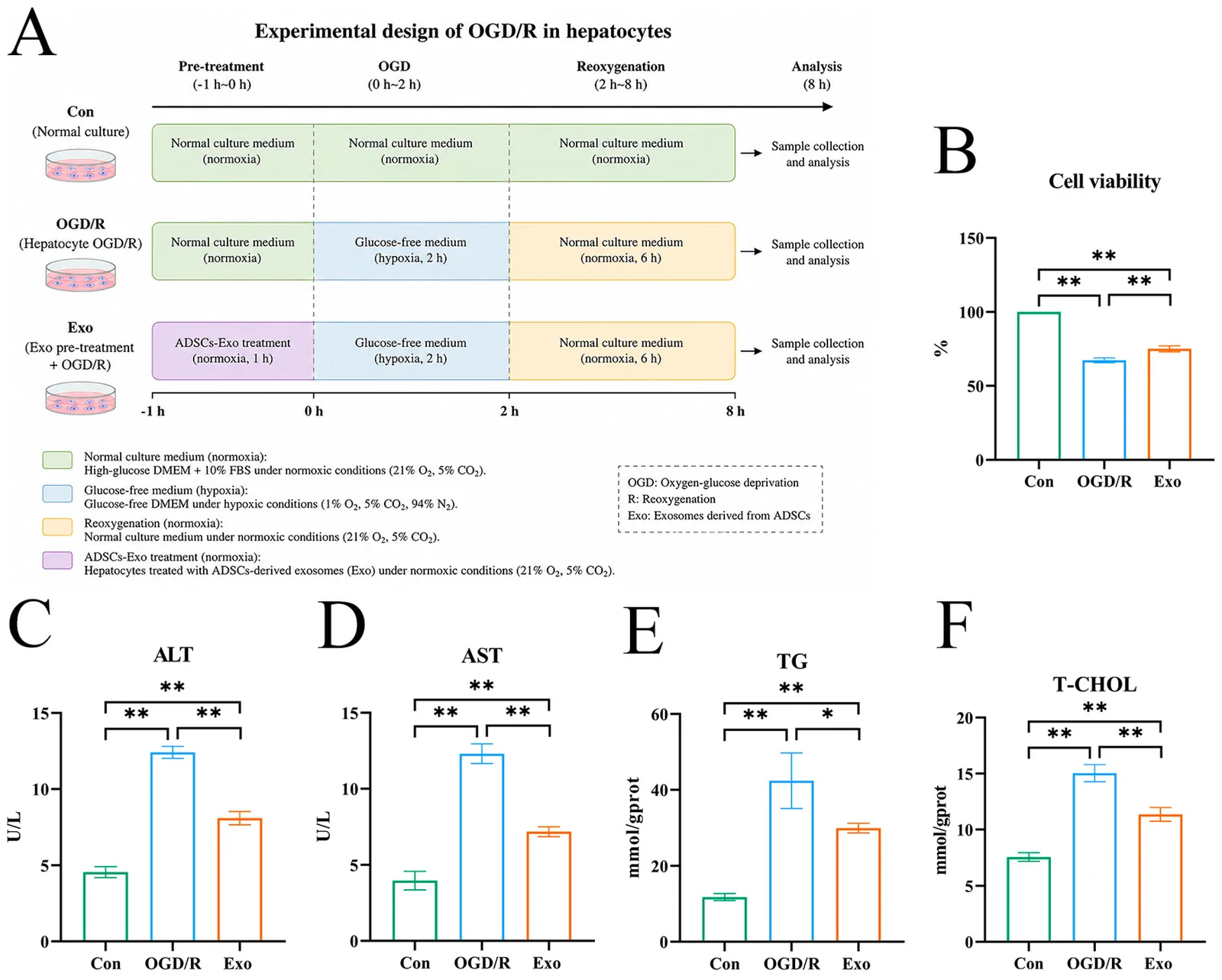
ADSCs-Exo attenuate OGD/R-induced hepatocyte injury. (**A**) Experimental design of OGD/R and ADSC-Exo treatment. (**B**) Hepatocyte viability. (**C**) ALT levels. (**D**) AST levels. (**E**) TG levels. (**F**) T-CHOL levels. The data are expressed as the mean ± SD. * *p* < 0.05, ** *p* < 0.01, and *n* = 3 independent experiments.

To evaluate the protective effects of ADSCs-Exo against OGD/R-induced hepatocyte injury, an in vitro OGD/R model was established. As shown in Figure 1A, hepatocytes were subjected to 2 h of oxygen–glucose deprivation followed by 6 h of reoxygenation under normal culture conditions. In the ADSC-Exo treatment group, hepatocytes were pretreated with ADSCs-Exo for 1 h before OGD and subsequently subjected to the same OGD/R procedure [34]. Thus, the ADSC-Exo treatment group represents OGD/R+ADSCs-Exo rather than ADSC-Exo treatment under non-injured conditions.

Compared with the Con group, OGD/R treatment significantly reduced hepatocyte viability (*p* = 0.0001), whereas ADSC-Exo pretreatment significantly restored cell viability compared with the OGD/R group (*p* = 0.0016) (Figure 1B). In addition, ALT and AST levels were significantly increased following OGD/R treatment (both *p* = 0.0001), while ADSC-Exo pretreatment markedly reduced the OGD/R-induced elevations in ALT and AST levels (both *p* = 0.0001) (Figure 1C,D). Further analysis showed that TG and T-CHOL levels were significantly elevated in the OGD/R group compared with the Con group (*p* = 0.0001 and 0.0003), indicating disturbed lipid metabolism following OGD/R injury. ADSCs-Exo pretreatment significantly reduced TG levels (*p* = 0.0282) and T-CHOL levels (*p* = 0.0008) compared with the OGD/R group (Figure 1E,F).

Collectively, these findings show that ADSCs-Exo pretreatment was associated with attenuation of OGD/R-induced injury-related changes relative to the OGD/R group. Because no non-injured ADSC-Exo-only group was included, these experiments do not determine the independent effects of ADSCs-Exo on hepatocytes under baseline conditions.

### 3.4. ADSC-Exo Treatment Is Associated with Attenuation of OGD/R-Induced Copper-Associated, Ferroptosis-Related, and Mitochondrial Alterations in Hepatocytes

OGD/R markedly induced hepatocyte injury, whereas ADSC-Exo treatment was associated with attenuation of these injury-related changes. To further characterize the biological alterations associated with this response, copper homeostasis and cuproptosis-related markers, mitochondrial function, and ferroptosis-related parameters were examined.

**Figure 2:**
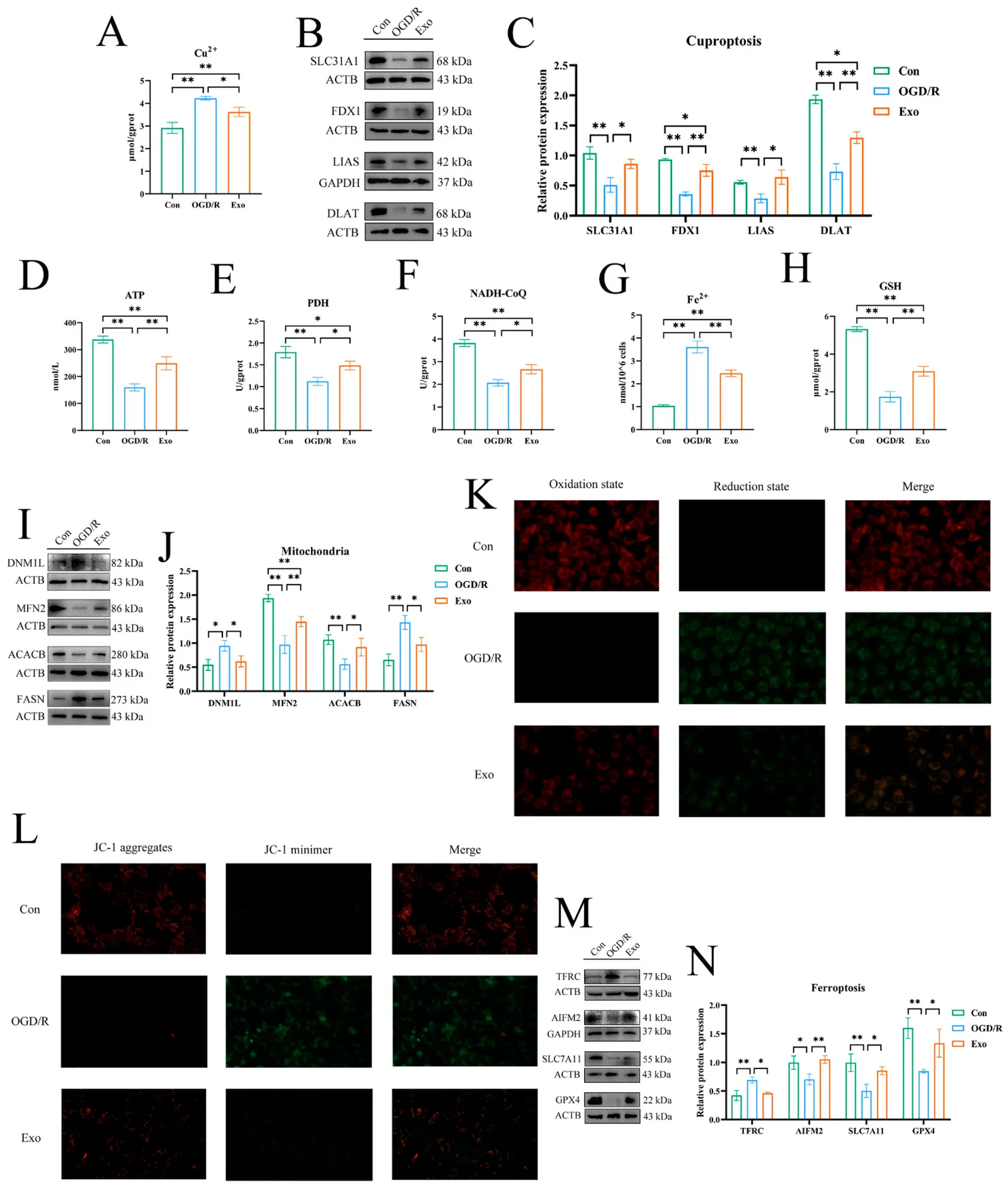
ADSCs-Exo attenuate OGD/R-induced copper-associated alterations, ferroptosis-related changes, and mitochondrial dysfunction in hepatocytes. (**A**) Intracellular Cu^2+^ levels. (**B**) Western blot analysis of cuproptosis-related proteins. (**C**) Quantification of SLC31A1, FDX1, LIAS, and DLAT. (**D**) ATP levels. (**E**) PDH activity. (**F**) NADH-CoQ activity. (**G**) Intracellular Fe^2+^ levels. (**H**) GSH levels. (**I**) Western blot analysis of mitochondrial dynamics- and lipid metabolism-related proteins. (**J**) Quantification of DNM1L, MFN2, ACACB, and FASN. (**K**) Cellular redox status. (**L**) Mitochondrial membrane potential assessed by JC-1 staining; (**M**) Western blot analysis of ferroptosis-related proteins; (**N**) quantification of TFRC, AIFM2, SLC7A11, and GPX4. The data are expressed as the mean ± SD. * *p* < 0.05, ** *p* < 0.01, and *n* = 3 independent experiments.

Compared with the Con group, intracellular Cu^2+^ levels were significantly increased following OGD/R treatment (*p* = 0.0003), whereas ADSC-Exo treatment significantly reduced Cu^2+^ accumulation (*p* = 0.0177) (Figure 2A). Meanwhile, OGD/R significantly decreased the protein expression of SLC31A1, FDX1, LIAS, and DLAT (*p =* 0.0017, 0.0001, 0.0169 and 0.0001), whereas ADSC-Exo treatment significantly reversed these changes (*p =* 0.0136, 0.0006, 0.0045 and 0.0012) (Figure 2B,C), indicating that OGD/R induced marked alterations in copper homeostasis and cuproptosis-related molecular events. Assessment of mitochondrial function showed that OGD/R significantly reduced ATP, PDH, and NADH-CoQ levels compared with the Con group (*p =* 0.0001, 0.0007 and 0.0001). ADSC-Exo treatment significantly increased ATP levels (*p* = 0.002) and restored PDH and NADH-CoQ levels (*p* = 0.0148 and 0.0117) (Figure 2D–F). In parallel, OGD/R significantly upregulated DNM1L (*p* = 0.0124) and FASN (*p* = 0.0011), while downregulating MFN2 and ACACB (*p* = 0.0002 and 0.0085). ADSC-Exo treatment significantly reversed the OGD/R-induced changes in DNM1L (*p* = 0.0296), MFN2 (*p* = 0.0094), ACACB (*p* = 0.0406), and FASN (*p* = 0.016) (Figure 2I,J). Furthermore, fluorescence-based assessment of cellular redox status and JC-1 staining showed that OGD/R disrupted cellular redox homeostasis and reduced mitochondrial membrane potential, whereas ADSCs-Exo treatment markedly ameliorated these abnormalities (Figure 2K,L). With respect to ferroptosis-related changes, OGD/R significantly increased intracellular Fe^2+^ levels and decreased GSH levels (both *p* = 0.0001), whereas ADSC-Exo treatment significantly reduced Fe^2+^ accumulation and restored GSH levels (*p* = 0.0004 and 0.001) (Figure 2G,H). Further analysis showed that OGD/R significantly upregulated TFRC expression (*p* = 0.0036) and downregulated AIFM2 (*p* = 0.0209), SLC7A11 (*p* = 0.0053), and GPX4 (*p* = 0.0047). ADSCs-Exo treatment significantly reduced TFRC expression (*p* = 0.0082) and restored the expression of AIFM2 (*p* = 0.0094), SLC7A11 (*p* = 0.0245), and GPX4 (*p* = 0.034) (Figure 2M,N).

Collectively, OGD/R was associated with copper accumulation, alterations in cuproptosis-related markers, ferroptosis-related changes, and marked mitochondrial dysfunction. Relative to the OGD/R group, ADSC-Exo treatment was associated with partial or substantial attenuation of these injury-related abnormalities. These findings indicate that, within the OGD/R injury context examined here, ADSC-Exo treatment is associated with coordinated improvement of copper-associated, ferroptosis-related, and mitochondrial parameters. However, because an ADSC-Exo-only non-injured group was not included, these data do not distinguish injury-dependent rescue effects from any potential baseline effects of ADSCs-Exo on otherwise uninjured hepatocytes.

### 3.5. Pharmacological Modulation of Copper Homeostasis Is Accompanied by Changes in Ferroptosis-Related Responses During OGD/R-Induced Hepatocyte Injury

The preceding results show that OGD/R simultaneously induced copper dyshomeostasis, alterations in cuproptosis-related markers, mitochondrial dysfunction, and ferroptosis-related changes in hepatocytes, whereas ADSCs-Exo ameliorated these abnormalities. To further assess the potential relationship between these processes, a bidirectional pharmacological intervention strategy was employed. TTM was used to reduce intracellular copper loading, whereas Cu(II)-Elesclomol was used to increase copper loading.

**Figure 3:**
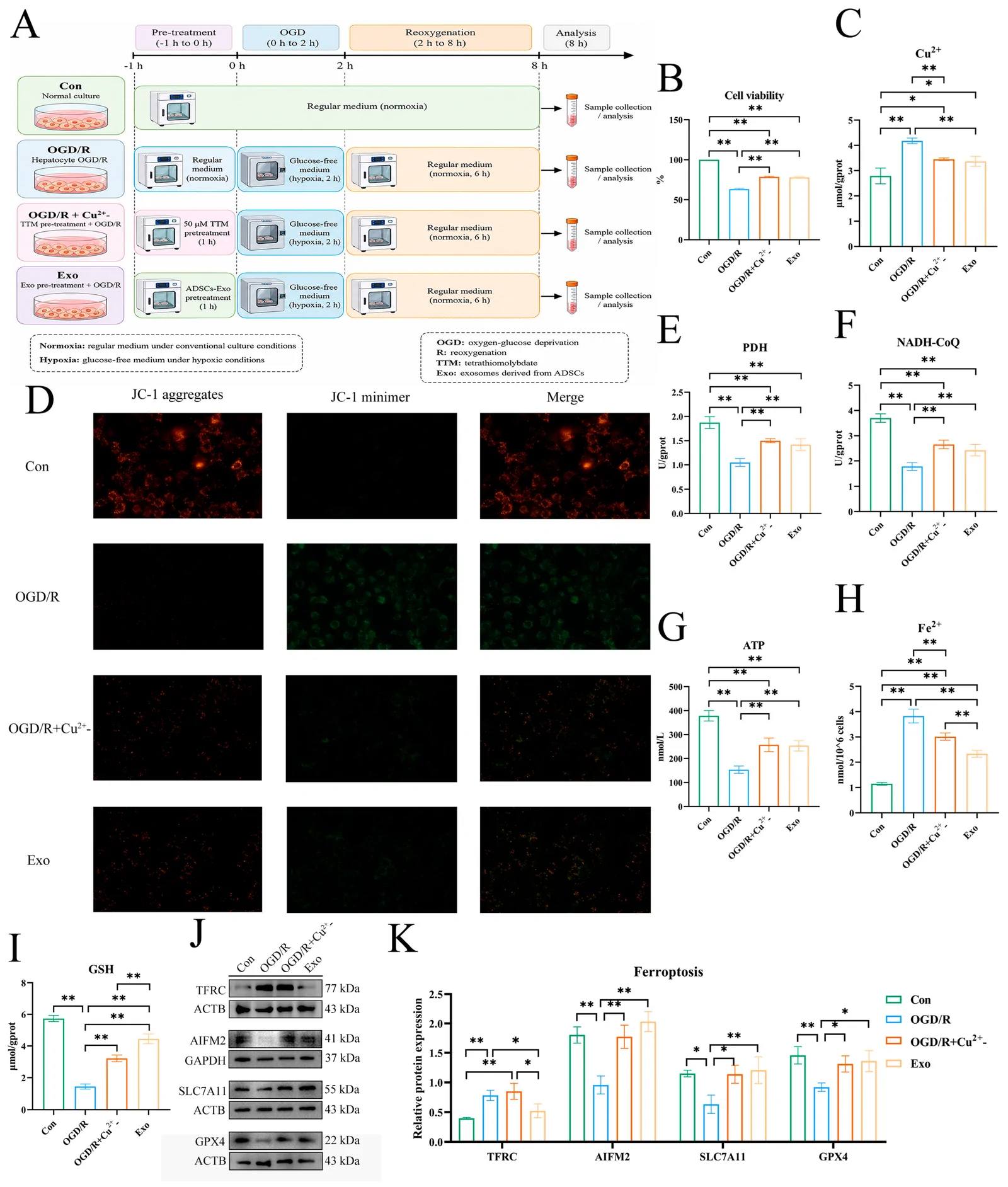
ADSC-Exo and TTM treatment attenuate OGD/R-induced mitochondrial dysfunction and ferroptosis-associated alterations in hepatocytes. (**A**) Experimental design of OGD/R, TTM, and ADSC-Exo treatments. (**B**) Hepatocyte viability. (**C**) Intracellular Cu^2+^ levels. (**D**) Mitochondrial membrane potential assessed by JC-1 staining. (**E**) PDH activity. (**F**) NADH-CoQ activity. (**G**) ATP levels. (**H**) Intracellular Fe^2+^ levels. (**I**) GSH levels. (**J**) Representative Western blot images of ferroptosis-related proteins. (**K**) Quantification of TFRC, AIFM2, SLC7A11, and GPX4 protein expression. The data are expressed as the mean ± SD. * *p* < 0.05, ** *p* < 0.01, and *n* = 3 independent experiments.

First, TTM treatment significantly reduced intracellular Cu^2+^ levels compared with the OGD/R group (*p* = 0.0073), accompanied by varying degrees of restoration in the expression of the cuproptosis-related markers SLC31A1, FDX1, LIAS, and DLAT (*p* = 0.0486, 0.0378, 0.0453, and 0.0224) (Figure 3C; Figure S2A,B), indicating that TTM attenuated OGD/R-induced copper accumulation and associated alterations in cuproptosis-related markers. Meanwhile, TTM significantly increased cell viability (*p* = 0.0001) and reduced ALT, AST, T-CHOL, and TG levels (*p* = 0.0001, 0.0001, 0.0009, and 0.0005) (Figure 3B; Figure S2C,E). With respect to mitochondrial function, TTM significantly restored PDH, NADH-CoQ, and ATP levels (*p* = 0.0021, 0.0016, and 0.002), improved mitochondrial membrane potential and cellular redox status, and reversed the abnormal expression of DNM1L, MFN2, ACACB, and FASN (*p* = 0.0098, 0.0235, 0.0287, and 0.0091) (Figure 3D–G; Figure S2D,F,G). TTM-mediated reduction in copper-associated alterations was also accompanied by significantly decreased intracellular Fe^2+^ levels and increased GSH levels (*p* = 0.0018 and 0.0001), together with restoration of AIFM2, SLC7A11, and GPX4 expression (*p* = 0.0014, 0.0192, and 0.0359) (Figure 3H–K). Within the OGD/R injury context, ADSCs-Exo treatment was associated with an overall improvement pattern similar to that observed following TTM treatment, including attenuation of copper-associated alterations, mitochondrial dysfunction, and ferroptosis-related abnormalities relative to the corresponding injured groups (Figure 3; Figure S2).

These findings indicate that pharmacological reduction in intracellular copper loading and associated changes in cuproptosis-related markers was accompanied by attenuation of ferroptosis-related responses. Importantly, because ADSCs-Exo were evaluated only in OGD/R-injured hepatocytes, these comparisons reflect treatment-associated changes within the injury setting and do not establish the independent effects of ADSCs-Exo under non-injured conditions.

**Figure 4:**
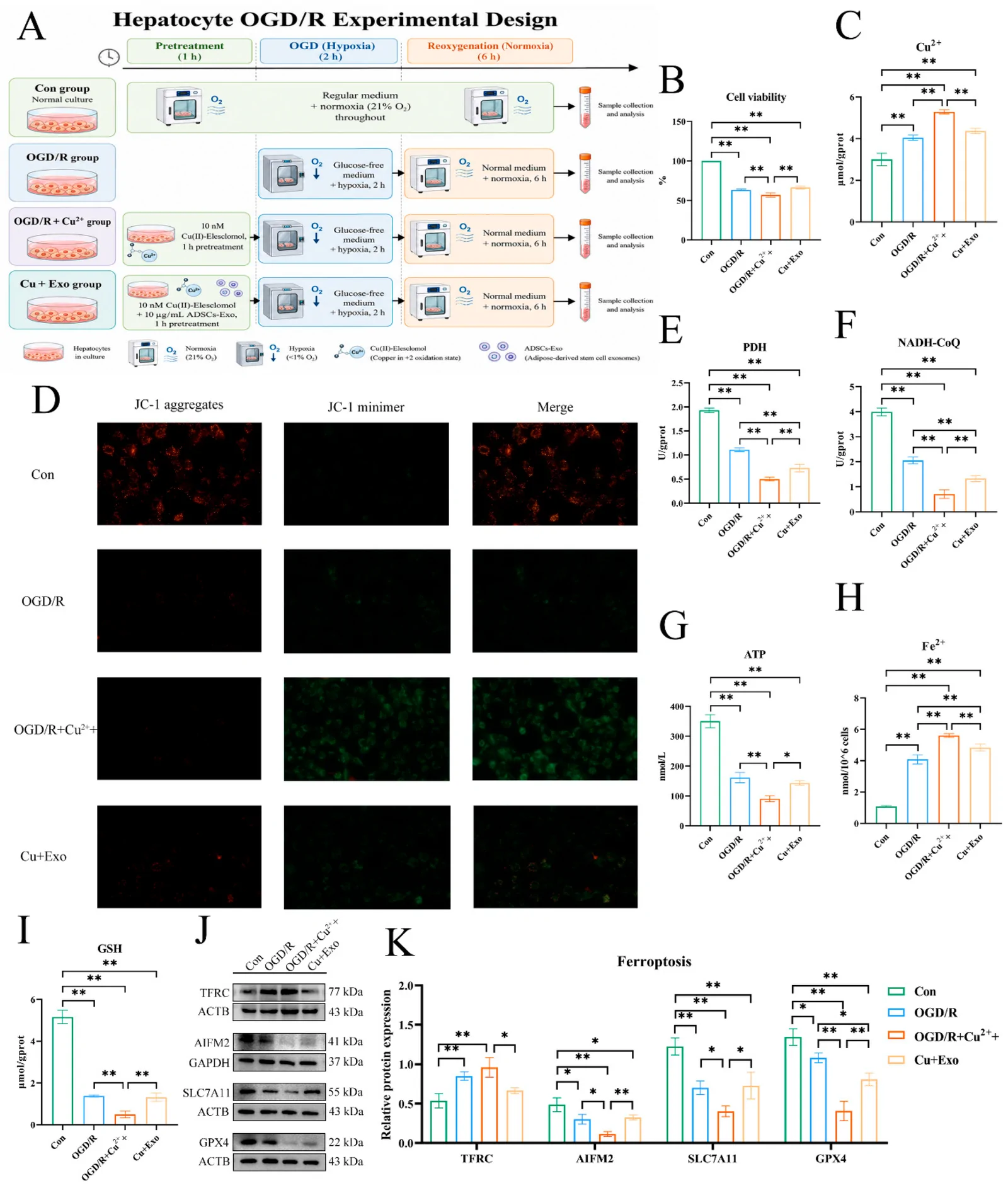
Cu(II)-Elesclomol treatment aggravates OGD/R-induced ferroptosis-associated alterations and mitochondrial dysfunction, which are attenuated by ADSCs-Exo. (**A**) Experimental design of OGD/R, Cu(II)-Elesclomol, and ADSC-Exo treatments. (**B**) Hepatocyte viability. (**C**) Intracellular Cu^2+^ levels. (**D**) Mitochondrial membrane potential assessed by JC-1 staining. (**E**) PDH activity. (**F**) NADH-CoQ activity. (**G**) ATP levels. (**H**) Intracellular Fe^2+^ levels. (**I**) GSH levels. (**J**) Representative Western blot images of ferroptosis-related proteins. (**K**) Quantification of TFRC, AIFM2, SLC7A11, and GPX4 protein expression. The data are expressed as the mean ± SD. * *p* < 0.05, ** *p* < 0.01, and *n* = 3 independent experiments.

To further determine whether increased copper loading was accompanied by aggravated ferroptosis-associated responses, Cu(II)-Elesclomol was used to further increase intracellular copper levels under OGD/R conditions. Compared with the OGD/R group, Cu(II)-Elesclomol treatment significantly increased intracellular Cu^2+^ levels (*p* = 0.0001) and further decreased the expression of SLC31A1, FDX1, LIAS, and DLAT (*p* = 0.0133, 0.0096, 0.0176, and 0.0242) (Figure 4C; Figure S3A,B), indicating that Cu(II)-Elesclomol-induced copper overload further aggravated alterations in cuproptosis-related markers. Meanwhile, Cu(II)-Elesclomol treatment further reduced cell viability (*p* = 0.0054) and increased ALT, AST, T-CHOL, and TG levels (all *p* = 0.0001) compared with the OGD/R group (Figure 4B; Figure S3C,E). Mitochondrial functional analysis showed that PDH, NADH-CoQ, and ATP levels were further decreased (*p* = 0.0001, 0.0001, and 0.0019), accompanied by greater impairment of mitochondrial membrane potential and cellular redox homeostasis and further dysregulation of DNM1L, MFN2, ACACB, and FASN expression (*p* = 0.0108, 0.0035, 0.0023, and 0.0024) (Figure 4D–G; Figure S3D,F,G). Notably, increased copper loading further elevated intracellular Fe^2+^ levels and reduced GSH levels (*p* = 0.0001 and 0.0037), while the expression of the ferroptosis-protective proteins AIFM2, SLC7A11, and GPX4 was further decreased (*p* = 0.0171, 0.049, and 0.0001) (Figure 4H–K). These findings indicate that Cu(II)-Elesclomol-induced copper overload and the accompanying alterations in cuproptosis-related markers were associated with aggravated ferroptosis-related responses under OGD/R conditions. Importantly, within the OGD/R plus copper loading context, ADSC-Exo treatment was associated with attenuation of the Cu(II)-Elesclomol-induced abnormalities relative to the corresponding copper loading group. In particular, cell viability, PDH, NADH-CoQ, ATP, Fe^2+^, and GSH levels were significantly improved (*p* = 0.0004, 0.0041, 0.0043, 0.0111, 0.0055, and 0.0058, respectively) (Figure 4; Figure S3).

Collectively, pharmacological reduction in intracellular copper loading with TTM attenuated ferroptosis-related responses, whereas Cu(II)-Elesclomol-induced copper overload produced the opposite pattern. These observations are consistent with an association between copper-associated alterations and ferroptosis-related responses under OGD/R conditions. ADSC-Exo treatment was associated with attenuation of several abnormalities in both the OGD/R and OGD/R plus copper loading settings when compared with the corresponding injured groups. However, because no non-injured ADSC-Exo-only group was included, these results do not determine whether ADSCs-Exo independently alter copper homeostasis, mitochondrial function, or ferroptosis-related parameters under baseline physiological conditions. Accordingly, the effects of ADSCs-Exo described here should be interpreted specifically within the injury contexts examined.

### 3.6. Pharmacological Modulation of Ferroptosis-Associated Responses Is Accompanied by Changes in Copper Homeostasis and Cuproptosis-Related Markers During OGD/R-Induced Hepatocyte Injury

The preceding experiments showed that pharmacological manipulation of copper homeostasis was accompanied by corresponding changes in ferroptosis-related parameters. We therefore further examined whether pharmacological enhancement or suppression of ferroptosis-associated responses was accompanied by reciprocal changes in copper homeostasis and cuproptosis-related markers. RSL3 and PKUMDL-LC-101-D013 were used as pharmacological interventions to enhance and suppress ferroptosis-associated responses, respectively.

**Figure 5:**
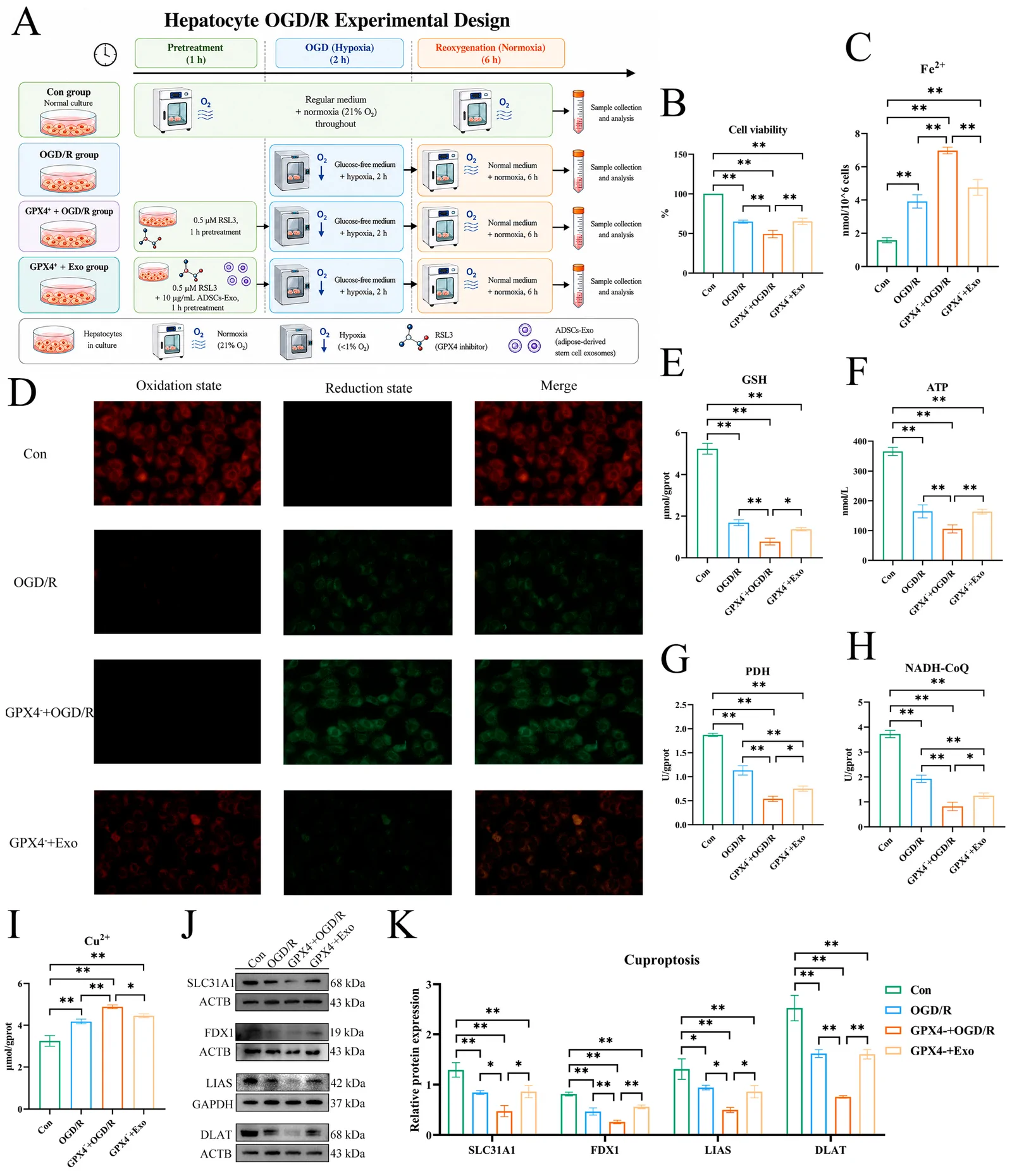
RSL3 treatment aggravates OGD/R-induced copper dyshomeostasis, alterations in cuproptosis-related markers, and mitochondrial dysfunction, which are ameliorated by ADSCs-Exo. (**A**) Experimental design of OGD/R, RSL3, and ADSC-Exo treatments. (**B**) Hepatocyte viability. (**C**) Intracellular Fe^2+^ levels. (**D**) Cellular redox status assessed by fluorescence staining. (**E**) GSH levels. (**F**) ATP levels. (**G**) PDH activity. (**H**) NADH-CoQ activity. (**I**) Intracellular Cu^2+^ levels. (**J**) Representative Western blot images of cuproptosis-related proteins. (**K**) Quantification of SLC31A1, FDX1, LIAS, and DLAT protein expression. The data are expressed as the mean ± SD. * *p* < 0.05, ** *p* < 0.01, and *n* = 3 independent experiments.

Compared with the OGD/R group, RSL3 treatment further reduced hepatocyte viability (*p* = 0.0016), significantly increased intracellular Fe^2+^ levels, and decreased GSH levels (*p* = 0.0001 and 0.0009). In parallel, the expression of the ferroptosis-protective proteins AIFM2, SLC7A11, and GPX4 was further reduced (*p* = 0.0083, 0.0189 and 0.0019) (Figure 5B,C,E; Figure S4D,E), indicating that RSL3 treatment further aggravated OGD/R-induced ferroptosis-associated alterations under the conditions used in this study. Consistently, ALT, AST, T-CHOL, and TG levels were further increased (all *p* = 0.0001) (Figure S4A,B), suggesting aggravated hepatocyte injury and lipid metabolic disturbance. With respect to mitochondrial function, RSL3 treatment further decreased ATP, PDH, and NADH-CoQ levels (*p* = 0.0061, 0.0004, and 0.0001). JC-1 staining showed a further reduction in mitochondrial membrane potential, accompanied by more pronounced dysregulation of DNM1L, MFN2, ACACB, and FASN expression (*p* = 0.0264, 0.0021, 0.0261, and 0.001) (Figure 5F–H; Figure S4C,F,G). Notably, RSL3 treatment further increased intracellular Cu^2+^ levels compared with the OGD/R group (*p* = 0.0023), while further decreasing the expression of the cuproptosis-related markers SLC31A1, FDX1, LIAS, and DLAT (*p* = 0.0151, 0.0029, 0.01, and 0.0003) (Figure 5I–K). These findings suggest that RSL3-induced enhancement of ferroptosis-associated responses was accompanied by aggravated copper dyshomeostasis and alterations in cuproptosis-related markers. Within the OGD/R plus RSL3 injury setting, ADSC-Exo treatment was associated with attenuation of several RSL3-associated abnormalities relative to the corresponding RSL3-treated group, including improvements in cell viability, ferroptosis-related parameters, and mitochondrial function, together with reduced Cu^2+^ accumulation and partial restoration of cuproptosis-related marker expression (Figure 5; Figure S4). Because ADSCs-Exo were not evaluated in non-injured hepatocytes, these findings should be interpreted as treatment-associated changes within the injured experimental context rather than as evidence of an independent baseline effect of ADSCs-Exo.

**Figure 6:**
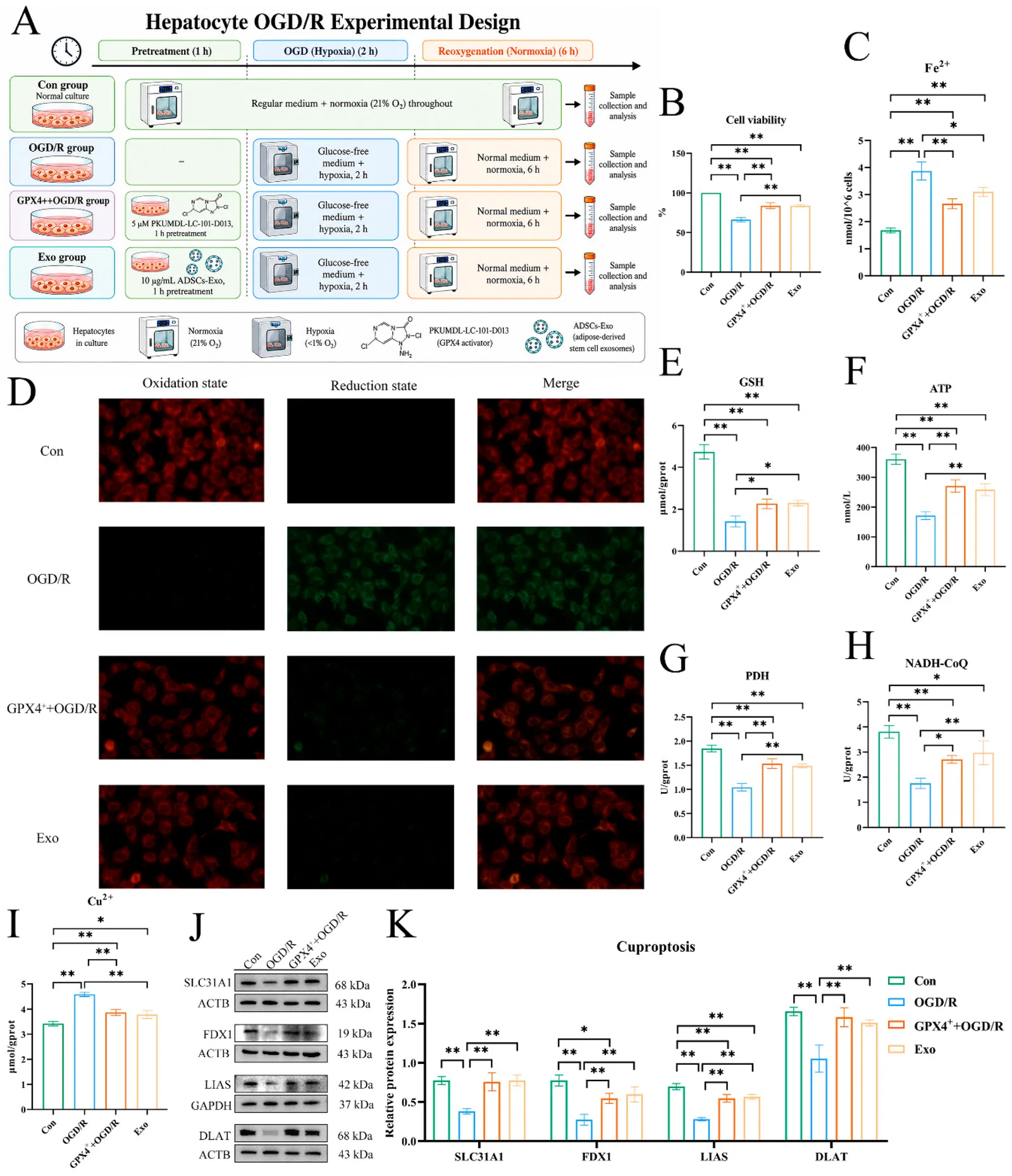
PKUMDL-LC-101-D013 treatment attenuates OGD/R-induced copper dyshomeostasis, alterations in cuproptosis-related markers, and mitochondrial dysfunction. (**A**) Experimental design of OGD/R, PKUMDL-LC-101-D013, and ADSC-Exo treatments. (**B**) Hepatocyte viability. (**C**) Intracellular Fe^2+^ levels. (**D**) Cellular redox status assessed by fluorescence staining. (**E**) GSH levels. (**F**) ATP levels. (**G**) PDH activity. (**H**) NADH-CoQ activity. (**I**) Intracellular Cu^2+^ levels. (**J**) Representative Western blot images of cuproptosis-related proteins. (**K**) Quantification of SLC31A1, FDX1, LIAS, and DLAT protein expression. The data are expressed as the mean ± SD. * *p* < 0.05, ** *p* < 0.01, and *n* = 3 independent experiments.

To further evaluate the effects of pharmacological suppression of ferroptosis-associated responses on copper homeostasis, PKUMDL-LC-101-D013 was used as a pharmacological intervention under OGD/R conditions. Compared with the OGD/R group, PKUMDL-LC-101-D013 treatment significantly increased hepatocyte viability (*p* = 0.0001), decreased intracellular Fe^2+^ levels (*p* = 0.0006), and increased GSH levels (*p* = 0.0153). In parallel, the expression of AIFM2, SLC7A11, and GPX4 was restored to varying degrees (*p* = 0.0006, 0.0183, and 0.0038) (Figure 6B,C,E; Figure S5D,E), indicating that PKUMDL-LC-101-D013 treatment attenuated OGD/R-induced ferroptosis-associated alterations under the conditions used in this study. Consistently, ALT, AST, T-CHOL, and TG levels were significantly reduced (*p* = 0.0001, 0.0043, 0.0121, and 0.0042) (Figure S5A,B). ATP and PDH levels were significantly increased compared with the OGD/R group (*p* = 0.0007 and 0.0002), while NADH-CoQ levels were also restored (*p* = 0.0182) (Figure 6F–H). PKUMDL-LC-101-D013 treatment also improved cellular redox homeostasis and mitochondrial membrane potential, accompanied by partial reversal of the abnormal expression of DNM1L, MFN2, ACACB, and FASN (*p* = 0.0002, 0.0327, 0.0378, and 0.0002) (Figure 6D; Figure S5C,F,G). Notably, PKUMDL-LC-101-D013 treatment significantly reduced intracellular Cu^2+^ levels compared with the OGD/R group (*p* = 0.0003) and restored the expression of the cuproptosis-related markers SLC31A1, FDX1, LIAS, and DLAT (*p* = 0.0011, 0.0098, 0.0001, and 0.0017) (Figure 6I–K). These findings indicate that pharmacological suppression of ferroptosis-associated responses was accompanied by attenuation of copper dyshomeostasis and alterations in cuproptosis-related markers.

Within the OGD/R injury setting, ADSCs-Exo treatment was associated with an improvement pattern similar to that observed following PKUMDL-LC-101-D013 treatment, including attenuation of ferroptosis-related abnormalities, improvement of mitochondrial function, reduced intracellular copper accumulation, and partial restoration of cuproptosis-related marker expression relative to the corresponding injured groups (Figure 6; Figure S5).

Collectively, RSL3 and PKUMDL-LC-101-D013 produced opposite pharmacological response patterns in ferroptosis-associated parameters, and these changes were accompanied by corresponding alterations in intracellular copper homeostasis and cuproptosis-related markers. Together with the preceding copper-directed pharmacological interventions, these findings are consistent with a potential reciprocal association between copper-associated alterations and ferroptosis-related responses under OGD/R conditions. ADSC-Exo treatment was associated with attenuation of several of these injury-related abnormalities when compared with the corresponding OGD/R-based injury groups. However, because a non-injured ADSC-Exo-only group was not included, the present experiments cannot determine the independent effects of ADSCs-Exo on ferroptosis-related parameters, copper homeostasis, or mitochondrial function under baseline conditions and cannot completely distinguish injury-specific rescue effects from potential baseline biological effects of exosome exposure. Therefore, the conclusions regarding ADSCs-Exo in this section are restricted to the OGD/R injury setting examined here.

### 3.7. ADSC-Exo Treatment Is Associated with Attenuation of Injury-Related Alterations in a Miniature Pig Model of Hepatic Ischemia–Reperfusion Injury

**Figure 7:**
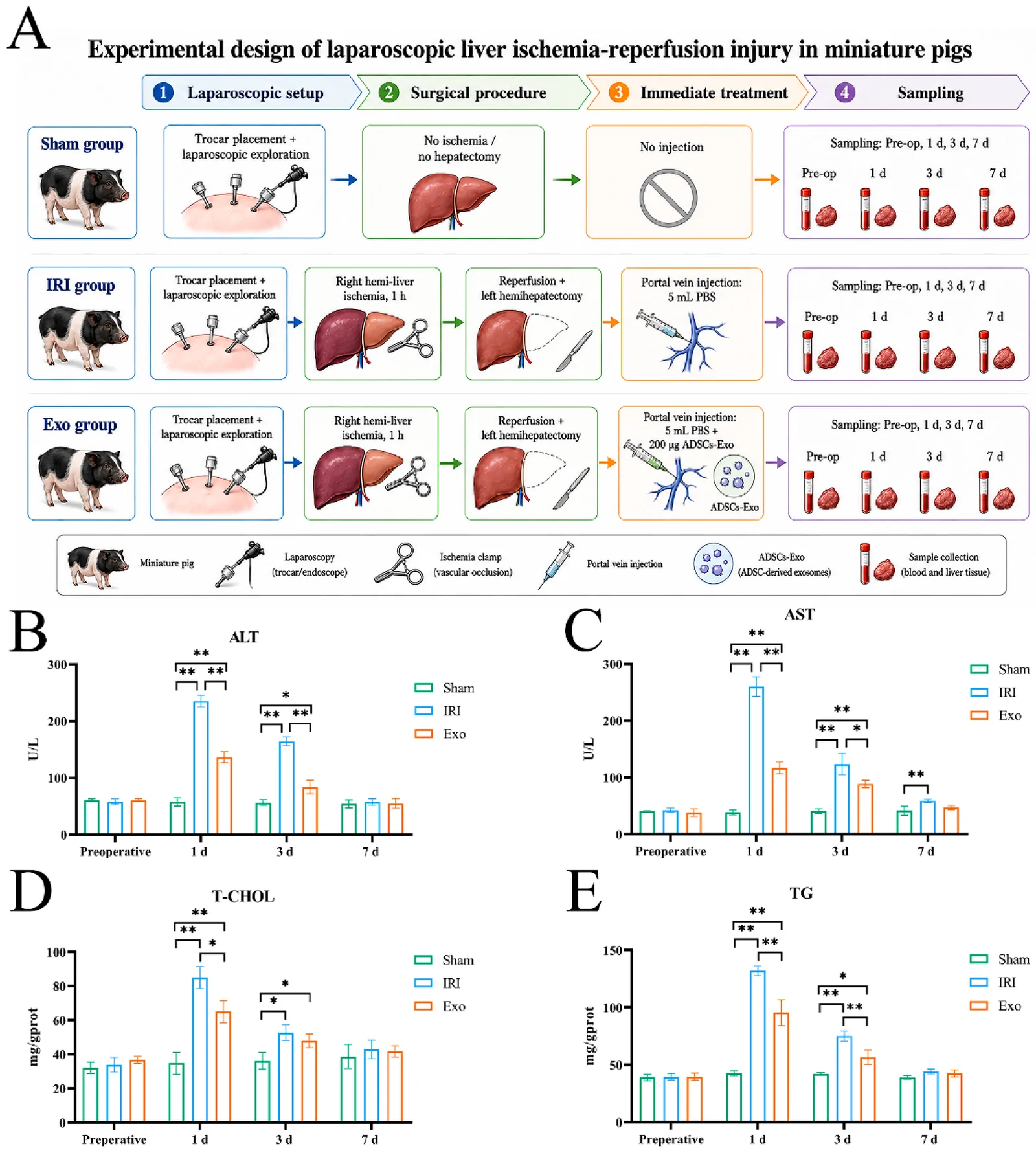
ADSCs-Exo alleviate hepatic ischemia–reperfusion injury in a miniature pig model. (**A**) Experimental design of laparoscopic hepatic IRI and ADSCs-Exo treatment in miniature pigs. (**B**) Serum ALT levels. (**C**) Serum AST levels. (**D**) Serum T-CHOL levels. (**E**) Serum TG levels. The data are expressed as the mean ± SD. * *p* < 0.05, ** *p* < 0.01, and *n* = 3 independent animals per group, randomly selected from the six animals included in each experimental group.

To determine whether the major response patterns observed in primary hepatocytes were also present in vivo, a laparoscopic miniature pig model of HIRI combined with partial hepatectomy was used. This model was employed to evaluate the effects of ADSCs-Exo on hepatic injury, lipid metabolic disturbance, copper-associated alterations involving cuproptosis-related markers, mitochondrial dysfunction, ferroptosis-related changes, and GPX4 homeostasis. Hepatic ischemia–reperfusion was induced laparoscopically and combined with partial hepatectomy to mimic clinically relevant hepatic IRI. Following model establishment, ADSCs-Exo were administered via the portal vein, and blood and liver tissue samples were collected before surgery and at 1, 3, and 7 days after surgery (Figure 7A). This in vivo model was used to further evaluate the effects of ADSCs-Exo on hepatocellular injury, lipid metabolic disturbance, hepatic copper homeostasis and cuproptosis-related markers, mitochondrial dysfunction, ferroptosis-related responses, and disruption of GPX4 homeostasis induced by hepatic IRI. Compared with the Sham group, serum ALT and AST levels, as well as T-CHOL and TG levels, were markedly increased after IRI. In the analytical subset of three animals per group, ADSC-Exo treatment was associated with significantly lower ALT levels than those in the IRI group at 1 and 3 days (*p* = 0.0001 and *p* = 0.0001, respectively) and lower AST levels at 1 and 3 days (*p* = 0.0001 and *p* = 0.0272, respectively). T-CHOL was reduced at 1 day (*p* = 0.0208), whereas TG was reduced at 1 and 3 days (*p* = 0.0017 and *p* = 0.0055, respectively) (Figure 7B–E). These results are consistent with attenuation of hepatocellular injury and lipid metabolic disturbance following ADSC-Exo treatment within the analyzed subset. However, given the analytical sample size of *n* = 3 animals per group, these findings should be interpreted cautiously and require confirmation in a larger cohort.

**Figure 8:**
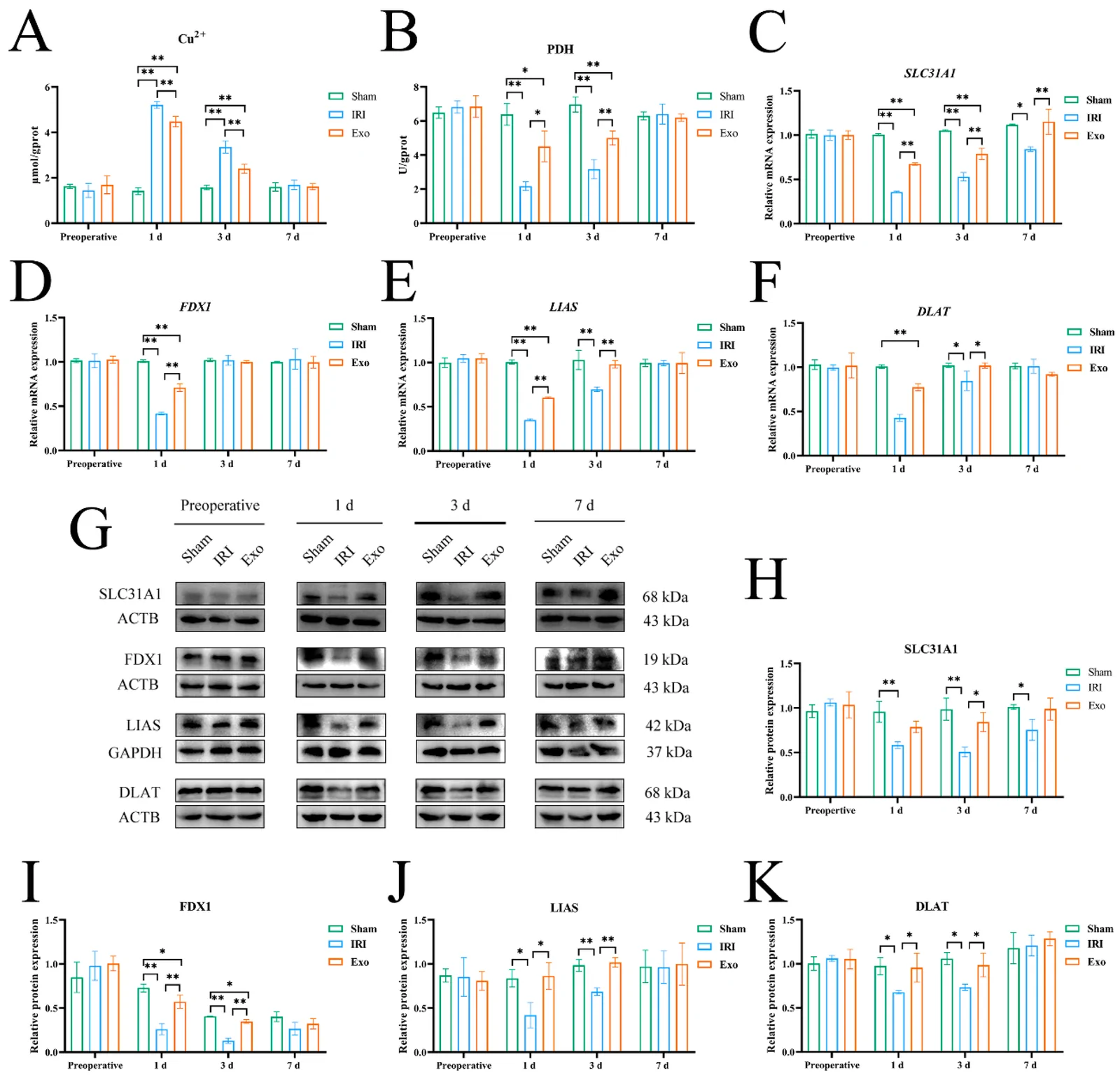
ADSC-Exo treatment is associated with attenuation of hepatic copper accumulation and alterations in cuproptosis-related markers during hepatic IRI in miniature pigs. (**A**) Hepatic Cu^2+^ levels. (**B**) Hepatic PDH activity. (**C**–**F**) Relative mRNA expression of SLC31A1, FDX1, LIAS, and DLAT, respectively. (**G**) Representative Western blot images of SLC31A1, FDX1, LIAS, and DLAT. (**H**–**K**) Quantification of SLC31A1, FDX1, LIAS, and DLAT protein expression, respectively. The data are expressed as the mean ± SD. * *p* < 0.05, ** *p* < 0.01, and *n* = 3 independent animals per group, randomly selected from the six animals included in each experimental group.

Further analysis showed that hepatic Cu^2+^ accumulation was markedly increased and PDH levels were reduced following IRI, accompanied by dysregulated expression of the cuproptosis-related molecules SLC31A1, FDX1, LIAS, and DLAT. ADSC-Exo treatment significantly reduced hepatic Cu^2+^ accumulation (day 1, *p* = 0.0055; day 3, *p* = 0.0029) and restored PDH levels (day 1, *p* = 0.0111; day 3, *p* = 0.0079), together with the expression of SLC31A1, FDX1, LIAS, and DLAT (Figure 8). These findings suggest that ADSCs-Exo were associated with attenuation of IRI-induced hepatic copper accumulation and alterations in cuproptosis-related markers in vivo.

**Figure 9:**
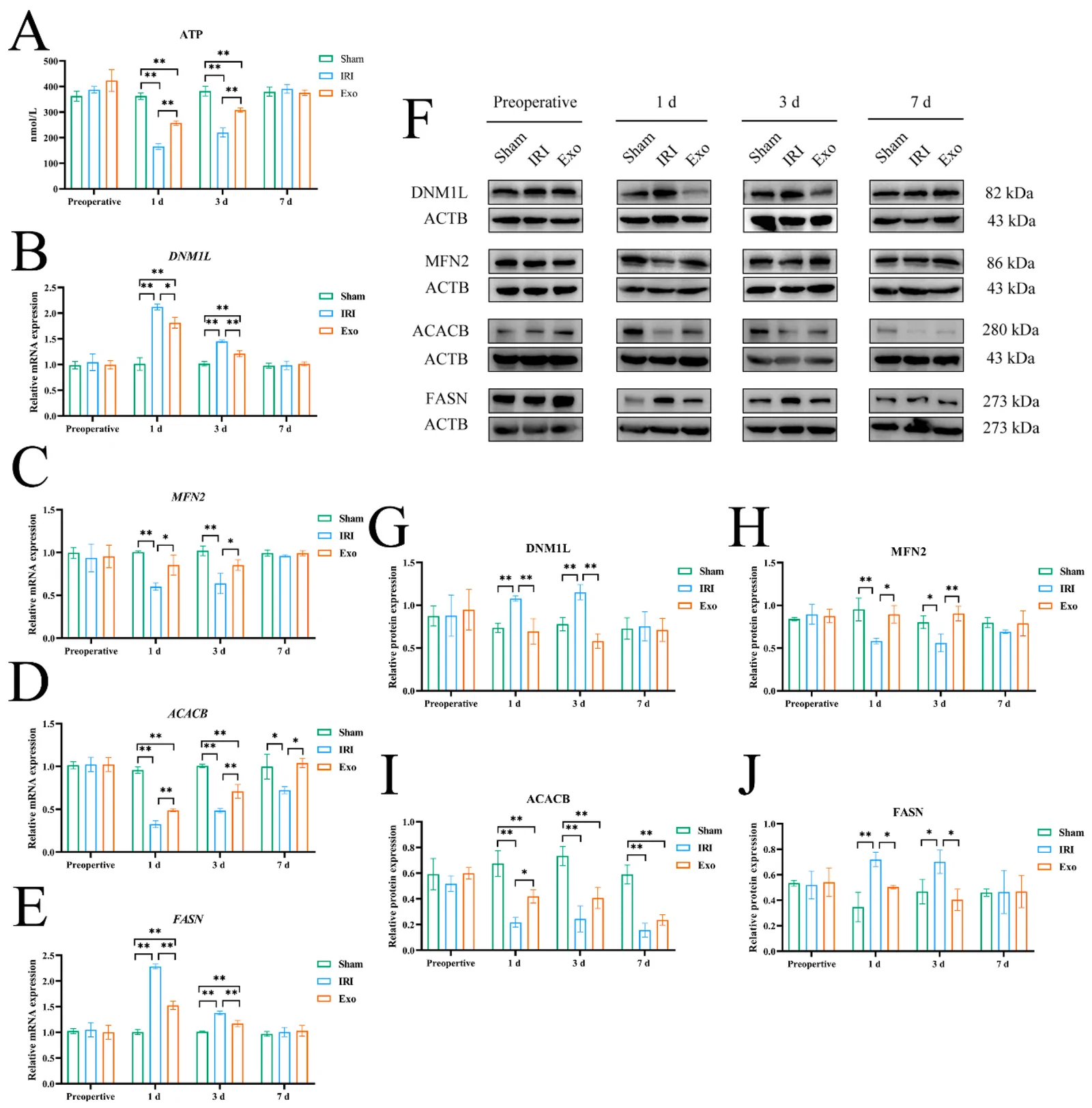
ADSCs-Exo ameliorate mitochondrial dysfunction and metabolic alterations in hepatic IRI in miniature pigs. (**A**) Hepatic ATP levels; (**B**–**E**) Relative mRNA expression of DNM1L, MFN2, ACACB, and FASN, respectively. (**F**) Representative Western blot images of DNM1L, MFN2, ACACB, and FASN. (**G**–**J**) Quantification of DNM1L, MFN2, ACACB, and FASN protein expression, respectively. The data are expressed as the mean ± SD. * *p* < 0.05, ** *p* < 0.01, and *n* = 3 independent animals per group, randomly selected from the six animals included in each experimental group.

Meanwhile, IRI decreased ATP levels and disrupted the expression of mitochondrial dynamics- and metabolism-related proteins, including DNM1L, MFN2, ACACB, and FASN. ADSC-Exo treatment significantly restored ATP levels (day 1, *p* = 0.0001; day 3, *p* = 0.0011) and reversed the abnormal expression of these mitochondrial proteins (Figure 9), further suggesting that ADSCs-Exo preserved mitochondrial energy metabolism and dynamic homeostasis.

**Figure 10:**
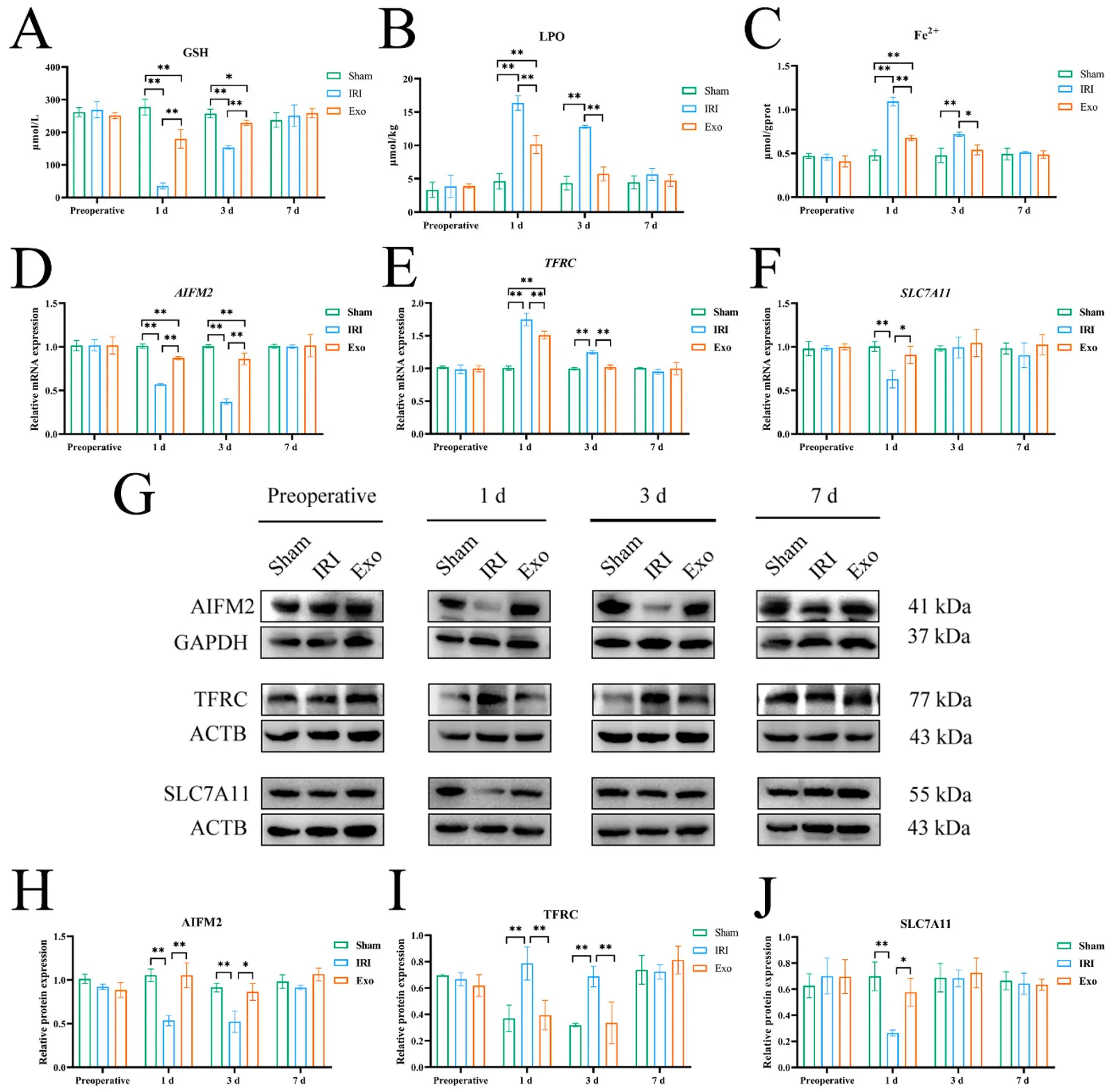
ADSCs-Exo attenuate ferroptosis-associated alterations in a miniature pig model of hepatic ischemia–reperfusion injury. (**A**) Hepatic GSH levels. (**B**) Hepatic LPO levels. (**C**) Hepatic Fe^2+^ levels. (**D**–**F**) Relative mRNA expression of *AIFM2*, *TFRC*, and *SLC7A11*, respectively. (**G**) Representative Western blot images of AIFM2, TFRC, and SLC7A11. (**H**–**J**) Quantification of AIFM2, TFRC, and SLC7A11 protein expression, respectively. The data are expressed as the mean ± SD. * *p* < 0.05, ** *p* < 0.01, and *n* = 3 independent animals per group, randomly selected from the six animals included in each experimental group.

Assessment of ferroptosis-related parameters showed that hepatic Fe^2+^ accumulation and LPO levels were increased, whereas GSH levels were decreased following IRI, accompanied by abnormal expression of the ferroptosis-related molecules AIFM2, SLC7A11, and TFRC. ADSC-Exo treatment significantly reduced hepatic Fe^2+^ accumulation (day 1, *p* = 0.0001; day 3, *p* = 0.0229) and LPO levels (day 1, *p* = 0.0018; day 3, *p* = 0.0001), while restoring GSH levels (day 1, *p* = 0.0005; day 3, *p* = 0.0001) and partially reversing the abnormal expression of AIFM2, SLC7A11, and TFRC (Figure 10). These findings suggest that ADSCs-Exo attenuated IRI-induced ferroptosis-associated alterations in vivo.

To explore whether alterations in GPX4 homeostasis were associated with changes in autophagy-related regulators, GPX4, TAX1BP1, and MAP1LC3B were examined. At 1 and 3 days after IRI, GPX4 mRNA and protein expression were markedly reduced, whereas TAX1BP1 expression was significantly increased and MAP1LC3B mRNA expression was elevated. ADSC-Exo treatment restored GPX4 expression while attenuating the abnormal changes in TAX1BP1 and MAP1LC3B (Figure S6A–F). Immunofluorescence staining further showed corresponding alterations in the expression patterns and distribution of GPX4, TAX1BP1, and MAP1LC3B following IRI, which were partially normalized by ADSC-Exo treatment (Figure S6G). These findings indicate an association between ADSC-Exo-associated preservation of GPX4 homeostasis and changes in TAX1BP1/MAP1LC3B-related autophagy markers. However, because autophagic flux, GPX4 turnover, and direct TAX1BP1–GPX4 interaction were not assessed, these observations should be regarded as hypothesis-generating evidence rather than mechanistic proof. Specifically, they support the hypothesis that TAX1BP1/MAP1LC3B-associated autophagic processes may contribute to the regulation of GPX4 homeostasis during HIRI, but this hypothesis remains untested in the present study.

Collectively, the in vivo experiments provided biological support for the major response patterns observed in vitro. Hepatic IRI was accompanied by hepatic copper accumulation, alterations in cuproptosis-related markers, mitochondrial dysfunction, ferroptosis-related changes, and reduced GPX4 expression, whereas ADSC-Exo treatment was associated with attenuation of these abnormalities relative to untreated IRI animals. The concomitant alterations in TAX1BP1 and MAP1LC3B further support the working hypothesis that autophagy-related processes may contribute to GPX4 homeostasis but do not establish a causal TAX1BP1-dependent GPX4 degradation mechanism. Together with the bidirectional pharmacological experiments in primary hepatocytes, these findings are consistent with a potential association among copper homeostasis, ferroptosis-related responses, mitochondrial dysfunction, and GPX4 homeostasis during HIRI, rather than establishing direct molecular interdependence or a causal hierarchy among these processes.

## 4. Discussion

The present study provides pharmacological evidence consistent with a potential association between copper-associated alterations and ferroptosis-related responses during HIRI. OGD/R-induced hepatocyte injury was accompanied by copper and iron dyshomeostasis, mitochondrial dysfunction, redox imbalance, alterations in several cuproptosis-related markers, and ferroptosis-associated changes. Bidirectional pharmacological interventions showed that manipulation of intracellular copper levels was accompanied by corresponding changes in ferroptosis-associated parameters, whereas pharmacological modulation of ferroptosis-associated responses produced reciprocal changes in copper homeostasis and cuproptosis-related markers. These findings are consistent with a potential reciprocal association between copper-associated and ferroptosis-related injury responses; however, they do not establish bona fide cuproptosis, direct bidirectional molecular interaction, or target-specific causality. Within the OGD/R and HIRI models examined here, ADSC-Exo treatment was associated with attenuation of abnormalities involving both processes relative to the corresponding injured controls.

Cuproptosis is closely linked to mitochondrial metabolism because excessive intracellular copper can disrupt lipoylated mitochondrial proteins and induce proteotoxic stress [11,40]. Beyond this specific cell death mechanism, copper homeostasis is essential for mitochondrial metabolism, redox regulation, and iron-related physiological processes, and its dysregulation has been implicated in various pathological conditions, including liver diseases [16,41]. In the present study, OGD/R was accompanied by intracellular Cu^2+^ accumulation, alterations in the cuproptosis-related markers SLC31A1, FDX1, LIAS, and DLAT, and impairment of mitochondrial function. Pharmacological reduction in copper loading with TTM attenuated these abnormalities, whereas increased copper loading with Cu(II)-Elesclomol produced the opposite pattern. These interventions were also accompanied by corresponding changes in Fe^2+^ accumulation, GSH levels, and ferroptosis-defense proteins, including AIFM2, SLC7A11, and GPX4. Previous studies have established several connections between copper dysregulation, mitochondrial dysfunction, and ferroptosis-related processes [14,16,18]. Furthermore, elesclomol has been reported to influence mitochondrial bioenergetic function [42]. Nevertheless, the present study did not directly assess the canonical biochemical hallmarks of cuproptosis, including aggregation of lipoylated mitochondrial proteins and loss of iron–sulfur cluster proteins. Therefore, the observed findings should be interpreted as copper-associated alterations involving cuproptosis-related markers rather than definitive evidence of bona fide cuproptotic cell death. In this context, the effects of TTM and Cu(II)-Elesclomol should primarily be interpreted as consequences of copper depletion and copper overload, respectively. Direct assessment of canonical cuproptosis hallmarks will be required in future studies to determine whether the copper-associated alterations observed here fulfill the biochemical criteria for bona fide cuproptosis.

The reciprocal pharmacological experiments further showed that manipulation of ferroptosis was accompanied by changes in copper homeostasis and cuproptosis-associated markers. RSL3 treatment increased Fe^2+^ accumulation and GSH depletion and was accompanied by greater Cu^2+^ accumulation and more pronounced alterations in SLC31A1, FDX1, LIAS, and DLAT, whereas pharmacological suppression of ferroptosis produced the opposite pattern. These observations suggest that ferroptosis-associated oxidative stress and mitochondrial injury may, in turn, influence cellular copper homeostasis. Because disturbances in copper homeostasis and ferroptosis-associated injury can both converge on mitochondrial dysfunction and redox imbalance [16,18,43,44], reciprocal amplification of cellular injury is biologically plausible. Nevertheless, the present pharmacological data are consistent with a potential reciprocal association between these processes rather than establishing functional interdependence or a direct molecular hierarchy.

This interpretation is particularly important because the bidirectional experiments were based on four pharmacological agents—TTM, Cu(II)-Elesclomol, RSL3, and PKUMDL-LC-101-D013. Although preliminary concentration– and time–response experiments were performed to establish empirical working conditions, these experiments were based on cell viability and were not designed to demonstrate molecular target engagement or compound specificity. Therefore, the present data do not independently establish that all effects produced by each compound were mediated exclusively through its presumed target or pathway. For example, the current experiments do not demonstrate that all effects of RSL3 at the selected concentration were specifically attributable to GPX4 inhibition. Consequently, compound-specific and off-target effects cannot be excluded. Genetic manipulation of key regulators such as FDX1 or GPX4, together with target-specific rescue experiments, will be necessary to establish direct molecular causality.

GSH/GPX4 homeostasis may represent an additional point of convergence between copper- and iron-associated injury responses. GSH is essential for GPX4-mediated suppression of lipid peroxidation [21,45] and also contributes to intracellular copper buffering [19,20]. Consistent with this concept, copper-directed interventions in the present study were accompanied by corresponding changes in GSH and GPX4. Previous studies have reported that copper can facilitate TAX1BP1-dependent autophagic degradation of GPX4 and thereby promote ferroptosis [14,46,47]. In our miniature pig model, HIRI was associated with reduced GPX4 expression, increased TAX1BP1 expression, and altered MAP1LC3B expression and immunofluorescence patterns, whereas ADSCs-Exo partially normalized these changes. Based on these coordinated expression patterns and the published literature, we propose the working hypothesis that TAX1BP1/MAP1LC3B-associated autophagic processes may contribute to the regulation of GPX4 homeostasis during HIRI. Importantly, this remains a hypothesis rather than a demonstrated mechanism. Autophagic flux was not directly assessed, GPX4 turnover was not measured, and a physical or functional TAX1BP1–GPX4 interaction was not established. Therefore, the present findings should be regarded as hypothesis-generating evidence and do not demonstrate TAX1BP1-dependent autophagic degradation of GPX4. Direct validation using lysosomal inhibitor-based autophagic flux assays, TAX1BP1 loss-of-function approaches, GPX4 turnover measurements, and protein interaction analyses will be required to test this hypothesis.

Within the injury models examined in the present study, ADSC-Exo treatment was consistently associated with attenuation of multiple OGD/R- and HIRI-related abnormalities relative to the corresponding injured controls. Previous studies, including our own, have shown that ADSCs-Exo alleviate HIRI through regulation of lipid metabolism, endoplasmic reticulum stress, mitochondrial homeostasis, inflammatory responses, and ferroptosis [28,29,30,31,32,33,35]. In the present experiments, ADSC-Exo treatment was associated with reduced copper accumulation, attenuation of alterations in cuproptosis-related markers, improved mitochondrial functional parameters, reduced iron accumulation and lipid peroxidation, preservation of GSH, and partial restoration of ferroptosis-defense systems. Importantly, similar treatment-associated patterns were observed in the miniature pig model combining hepatic ischemia–reperfusion with partial hepatectomy, providing large-animal support for the biological relevance of these findings. However, because neither a non-injured ADSC-Exo-only hepatocyte group nor a Sham + ADSC-Exo animal group was included, the present study cannot independently determine the biological effects of ADSCs-Exo under baseline conditions. The experimental design therefore does not allow for complete distinction between injury-context-dependent rescue effects and potential baseline effects of exosome exposure. Accordingly, the conclusions regarding ADSCs-Exo are restricted to the OGD/R and HIRI settings examined here, and no conclusions are drawn regarding their baseline physiological effects or safety in non-injured cells or tissues.

From a translational perspective, the use of a miniature pig model combining hepatic ischemia–reperfusion with partial hepatectomy provides a large-animal context in which the major injury-associated response patterns observed in vitro could be further evaluated in vivo. The treatment-associated attenuation of hepatocellular injury, mitochondrial dysfunction, copper-associated alterations, and ferroptosis-related abnormalities observed in this model may be relevant to hepatic ischemia–reperfusion encountered during liver transplantation and major hepatic resection, particularly during the reperfusion period [48,49,50,51,52,53,54]. Nevertheless, these observations remain preclinical and should not be interpreted as establishing therapeutic efficacy in clinical patients.

Several limitations should be considered when interpreting these findings. First, the proposed reciprocal association between copper-associated alterations and ferroptosis-related responses was inferred primarily from pharmacological modulation using TTM, Cu(II)-Elesclomol, RSL3, and PKUMDL-LC-101-D013. TTM and Cu(II)-Elesclomol directly alter intracellular copper availability; therefore, the corresponding experiments primarily establish the consequences of copper depletion or copper overload. Although SLC31A1, FDX1, LIAS, and DLAT were assessed as cuproptosis-related markers, canonical hallmarks of cuproptosis, including DLAT oligomerization, loss of lipoylated proteins, and loss of Fe–S cluster proteins, were not directly measured. Accordingly, these findings should be interpreted as copper-associated alterations involving cuproptosis-related markers rather than definitive evidence of bona fide cuproptosis. In addition, although preliminary concentration– and time–response screening was performed for the pharmacological agents, this screening was based on cell viability and was intended to define empirical working conditions rather than to establish molecular target engagement or compound specificity. Therefore, compound-specific and off-target effects cannot be excluded. Genetic manipulation of key regulators such as FDX1 or GPX4, together with target-specific rescue experiments and direct assessment of canonical cuproptosis hallmarks, will be required to establish molecular causality. Second, the involvement of TAX1BP1/MAP1LC3B-associated autophagy in GPX4 regulation should be regarded strictly as a working hypothesis. This hypothesis is based on coordinated expression and immunofluorescence patterns rather than direct demonstration of autophagic flux, GPX4 turnover, or TAX1BP1–GPX4 interaction. Therefore, no causal TAX1BP1-dependent GPX4 degradation mechanism can be established from the present data. Future studies incorporating lysosomal inhibitor-based autophagic flux assays, TAX1BP1 loss-of-function approaches, protein interaction analyses, and GPX4 turnover measurements will be required to test this hypothesis directly. Third, neither a non-injured ADSC-Exo-only group in the hepatocyte experiments nor a Sham + ADSC-Exo group in the miniature pig experiments was included. Consequently, although ADSC-Exo treatment was associated with attenuation of multiple OGD/R- and HIRI-induced abnormalities relative to the corresponding injured controls, the present study cannot independently determine the effects of ADSCs-Exo on normal hepatocytes or uninjured liver tissue. The experimental design therefore does not allow us to distinguish completely between injury context-dependent rescue effects and potential baseline biological effects of ADSC-Exo exposure. Accordingly, all conclusions regarding ADSCs-Exo have been restricted to the OGD/R and HIRI settings examined in this study, and no conclusions are drawn regarding their baseline physiological effects or safety in non-injured cells or tissues. Future studies incorporating non-injured ADSC-Exo-only controls will be required to address this question directly. Finally, although six miniature pigs were included in each experimental group, the biochemical and molecular endpoints reported in Figure 7, Figure 8, Figure 9 and Figure 10, including serum ALT and AST, were analyzed in a randomly selected subset of three animals per group. The animal cohort formed part of a broader research program involving multiple parallel analytical endpoints, and the available blood and tissue samples were distributed across these analyses. Although serum ALT and AST individually require only small sample volumes, they were measured within the same predefined analytical subset used for the other endpoints reported in this study. Samples from the remaining animals were allocated to other parallel analyses and have since been fully utilized, precluding retrospective measurement of ALT and AST in these animals. We acknowledge that this analytical design, particularly the use of *n* = 3 for the reported in vivo endpoints, limits statistical precision and generalizability. In addition, no formal a priori power calculation was performed. Therefore, the in vivo findings should be regarded as exploratory and require confirmation in larger, prospectively powered cohorts.

## 5. Conclusions

In conclusion, the present study provides pharmacological evidence consistent with a potential reciprocal association between copper-associated alterations and ferroptosis-related responses during OGD/R-induced hepatocyte injury. Pharmacological manipulation of intracellular copper homeostasis was accompanied by corresponding changes in ferroptosis-related parameters, whereas pharmacological modulation of ferroptosis-associated responses was accompanied by reciprocal alterations in copper homeostasis and cuproptosis-related markers. Mitochondrial dysfunction, redox imbalance, and impaired GSH/GPX4 homeostasis may represent potential points of convergence between these injury responses. Within the OGD/R and HIRI models examined here, ADSC-Exo treatment was associated with attenuation of copper accumulation, alterations in cuproptosis-related markers, mitochondrial dysfunction, iron accumulation, lipid peroxidation, GSH depletion, impairment of ferroptosis defense systems, and hepatocellular injury relative to the corresponding untreated injury groups. Because non-injured ADSC-Exo-only controls were not included, these findings do not establish the independent effects of ADSCs-Exo under baseline physiological conditions and should be interpreted specifically within the injury settings examined in this study. From a translational perspective, the treatment-associated attenuation observed in a large-animal model combining hepatic ischemia–reperfusion with partial hepatectomy may be relevant to liver injury encountered during liver transplantation and major hepatic resection; however, these findings remain preclinical. Moreover, because canonical hallmarks of cuproptosis were not directly assessed, the present results should be interpreted as evidence of copper-associated alterations involving cuproptosis-related markers rather than a definitive demonstration of bona fide cuproptosis. Further genetic, target-specific, cuproptosis-specific, and adequately powered studies will be required to establish causal molecular relationships and more precisely define the injury context-dependent effects of ADSCs-Exo.

## Figures and Tables

**Table 1:** Antibodies used for ADSC and ADSC-Exo identification.

Name	Producers	Catalog Number	Dilution
CD29	Abcam	ab21845	1:100
CD44	Abcam	ab95138	1:100
CD90	Abcam	ab124527	1:100
CD11b	Biolegend	301329	1:100
CD63	Abcam	ab231975	1:1000
CD81	Absin	abs126599	1:1000
TSG101	LSBio	LS-C408134	1:2000

**Table 2:** Gene-specific primers used for RT-qPCR and their corresponding NCBI RefSeq accession numbers.

Gene	Forward Primer Sequence (5′–3′)	Reverse Primer Sequence (5′–3′)	NCBI RefSeq Accession Number
*ACTB*	TCTGGCACCACACCTTCT	TGATCTGGGTCATCTTCTCAC	NM_001444420.1
*SLC31A1*	CACACAAAACAGTTGGGCAG	CACAGGTACCCGTTGTAGGT	NM_214100.3
*DLAT*	GATCGGCACTGATTCCCACA	TCACACCAATCACCTTGGGG	NM_213994.2
*FDX1*	TGGCTTGCTCTACCTGTCAC	CCCAGCCGTGATCTATCTGT	NM_214065.1
*LIAS*	TGTATGCCCCCGGGTATTTG	TCATCCCAGGTGCTCTTGTC	NM_001243462.1
*DNM1L*	AACAACAGGCAACTGGAGAGGAATG	TGGCGGGCATGATTGGAATTGG	XM_021092064.2
*MFN2*	CCACACCACCAACTGCTTCCTG	TCTTGACGCTCCTCTTCTCCTCTG	XM_021095369.1
*FASN*	TGGATCACTGCATAGACGGC	TGGTACACCTTCCCGCTTG	NM_001099930.1
*ACACB*	CCCGACCATGTTTGTCCTCA	GGTGAGGCGGTAACTGTTGA	NM_001206399.1
*TFRC*	ACCTCGCTTATTTTGGGCAGA	CTTTGAGCATTTGCCACCTTCT	NM_214001.1
*SLC7A11*	CTGGCATTTGGACGCTACA	CAACAGTTCCTCGGCACTAA	XM_084972876.1
*GPX4*	TACGGATTCTGGCCTTCCCT	AGTTCCATTTGATAGCATTTCCCAG	NM_214407.1
*AIFM2*	TGGCCATGGGGAGAAATGAC	GCCGCATGGTTTTCCAACTT	XM_084997207.1
*MAP1LC3B*	TTCTTCCTGTTAGTGAACGGACATAGC	ATCCATCTTCATCCTTCTCGCTTTCG	NM_001190290.1
*TAX1BP1*	TTCAGGCAACTCGGCAAGAA	ACTGCATCGGCTAACTGCTT	XM_021079294.2

**Table 3:** Antibodies used for Western blotting.

Name of Protein	Producers	Catalog Number	Dilution	Reactivity
ACTB	Proteintech	81115-1-RR	1:10000	Rabbit
GAPDH	Proteintech	60004-1-Ig	1:50000	Mouse
SLC31A1	BYabscience	BYab-0857	1:1000	Rabbit
DLAT	Abmart	T58125	1:1000	Rabbit
FDX1	Affinity	DF7950	1:1000	Rabbit
LIAS	Proteintech	11577-1-AP	1:1000	Mouse
DNM1L	ABclonal	A2586	1:1000	Rabbit
MFN2	Proteintech	12186-1-AP	1:5000	Rabbit
FASN	ABclonal	A19050	1:1000	Rabbit
ACACB	ABclonal	AP1410PM	1:1000	Rabbit
TFRC	BYabscience	BYab-17749	1:1000	Rabbit
SLC7A11	BYabscience	Byab-17924	1:1000	Rabbit
GPX4	HUABIO	ET1706-45	1:10000	Rabbit
TAX1BP1	GenulN	61175	1:1000	Rabbit

## Data Availability

The data used to support the findings of this study are available from the corresponding author upon request.

## Supplementary Materials

The following supporting information can be downloaded at: https://www.mdpi.com/article/10.3390/antiox15101335/s1: The supplementary materials contain Figures S1–S6, including preliminary concentration– and time–response screening of the pharmacological modulators and additional biochemical, molecular, and immunofluorescence analyses supporting the main findings. Figure S1. Preliminary concentration- and time-response screening of pharmacological modulators in primary hepatocytes under OGD/R conditions. (A) TTM, (B) Cu(II)-Elesclomol, (C) RSL3, or (D) PKUMDL-LC-101-D013 for 1, 6, or 12 h. Cell viability was evaluated using the CCK-8 assay. These preliminary experiments were performed to determine the working concentrations and exposure conditions used in the subsequent pharmacological intervention experiments. Data are presented as mean ± SD, *n* = 3 independent experiments. Figure S2. ADSCs-Exo and cuproptosis inhibition alleviate OGD/R-induced hepatocyte injury, mitochondrial dysfunction, and ferroptosis-associated alterations. (A) Representative western blot images of cuproptosis-related proteins. (B) Quantification of CTR1, FDX1, LIAS, and DLAT protein expression. (C) ALT and AST levels. (D) Cellular redox status assessed by fluorescence staining. (E) T-CHOL and TG levels. (F) Representative western blot images of mitochondrial dynamics- and lipid metabolism-related proteins. (G) Quantification of Drp1, MFN2, ACACB, and FASN protein expression. The data were expressed as mean ± SD. * 0.01 < *p* < 0.05, ** *p* < 0.01, *n* = 3 independent experiments. Figure S3. Enhanced cuproptosis aggravates OGD/R-induced hepatocyte injury, mitochondrial dysfunction, and ferroptosis-associated alterations, which are ameliorated by ADSCs-Exo. (A) Representative western blot images of cuproptosis-related proteins. (B) Quantification of CTR1, FDX1, LIAS, and DLAT protein expression. (C) ALT and AST levels. (D) Cellular redox status assessed by fluorescence staining. (E) T-CHOL and TG levels. (F) Representative western blot images of mitochondrial dynamics- and lipid metabolism-related proteins. (G) Quantification of Drp1, MFN2, ACACB, and FASN protein expression. The data were expressed as mean ± SD. * 0.01 < *p* < 0.05, ** *p* < 0.01, *n* = 3 independent experiments. Figure S4. Ferroptosis enhancement aggravates OGD/R-induced hepatocyte injury, mitochondrial dysfunction, and cuproptosis-associated alterations, which are ameliorated by ADSCs-Exo. (A) ALT and AST levels. (B) T-CHOL and TG levels. (C) Mitochondrial membrane potential assessed by JC-1 staining. (D) Representative western blot images of ferroptosis-related proteins. (E) Quantification of AIFM2, SLC7A11, and GPX4 protein expression. (F) Representative western blot images of mitochondrial dynamics- and lipid metabolism-related proteins. (G) Quantification of Drp1, MFN2, ACACB, and FASN protein expression. The data were expressed as mean ± SD. * 0.01 < *p* < 0.05, ** *p* < 0.01, *n* = 3 independent experiments. Figure S5. ADSCs-Exo and ferroptosis suppression alleviate OGD/R-induced hepatocyte injury, mitochondrial dysfunction, and cuproptosis-associated alterations. (A) ALT and AST levels. (B) T-CHOL and TG levels. (C) Mitochondrial membrane potential assessed by JC-1 staining. (D) Representative western blot images of ferroptosis-related proteins. (E) Quantification of AIFM2, SLC7A11, and GPX4 protein expression. (F) Representative western blot images of mitochondrial dynamics- and lipid metabolism-related proteins. (G) Quantification of Drp1, MFN2, ACACB, and FASN protein expression. The data were expressed as mean ± SD. * 0.01 < *p* < 0.05, ** *p* < 0.01, *n* = 3 independent experiments. Figure S6 ADSCs-Exo restore GPX4 expression and ameliorate TAX1BP1/MAP1LC3B-associated autophagic alterations following hepatic ischemia-reperfusion injury in miniature pigs. (A–C) Relative mRNA expression of GPX4, TAX1BP1, and MAP1LC3B, respectively. (D) Representative western blot images of GPX4 and TAX1BP1. (E, F) Quantification of GPX4 and TAX1BP1 protein expression, respectively. (G) Representative immunofluorescence images of GPX4, TAX1BP1, and MAP1LC3B in liver tissues. The data were expressed as mean ± SD. * 0.01 < *p* < 0.05, ** *p* < 0.01, *n* = 3 independent animals per group, randomly selected from the six animals included in each experimental group. Table S1. ACTB Ct values.

## Abbreviations

The following abbreviations are used in this manuscript:

ADSCs-Exo	adipose-derived mesenchymal stem cell-derived exosomes
ALT	alanine transaminase
ANOVA	analysis of variance
AST	aspartate transaminase
GSH	glutathione
HIRI	hepatic ischemia–reperfusion injury
LPO	lipid peroxidation
MMP	mitochondrial membrane potential
OGD/R	Oxygen–glucose deprivation/reoxygenation
PDH	pyruvate dehydrogenase
T-CHOL	total cholesterol
TG	triglycerides
