# Supplementary material for: ADSCs-Exo Attenuate HIRI in Miniature Pigs: Pharmacological Evidence for a Potential Association Between Copper-Associated Alterations and Ferroptosis-Related Responses
Source: Antioxidants (Basel). 2026 Oct 16;15(10):1335. doi: 10.3390/antiox15101335 (PMC13701778; doi:10.3390/antiox15101335)
Supplement: Supplementary file 1 [file antioxidants-15-01335-s001.zip › Supplementary figures.pdf]

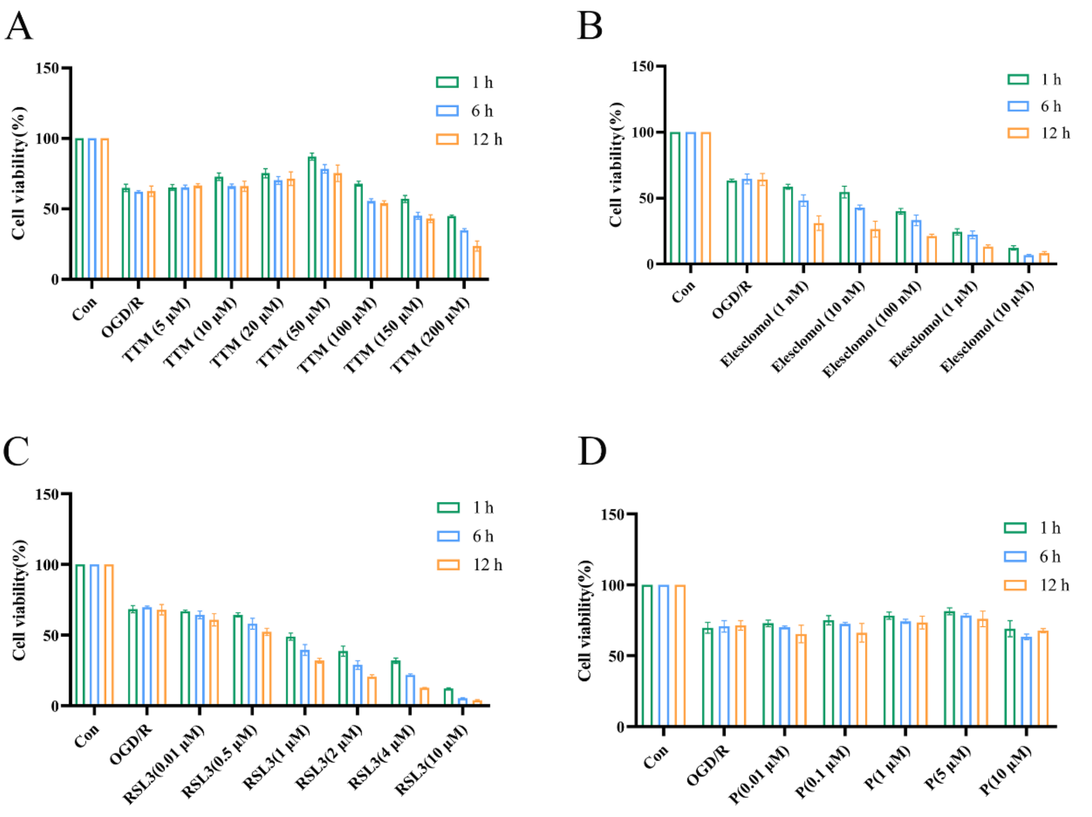

Figure S1. Preliminary concentration- and time-response screening of pharmacological modulators in primary hepatocytes under OGD/R conditions.

Note: (A) TTM, (B) Cu(II)-Elesclomol, (C) RSL3, or (D) PKUMDL-LC-101-D013 for 1, 6, or 12 h. Cell viability was evaluated using the CCK-8 assay. These preliminary experiments were performed to determine the working concentrations and exposure conditions used in the subsequent pharmacological intervention experiments. Data are presented as mean ± SD, n = 3 independent experiments.

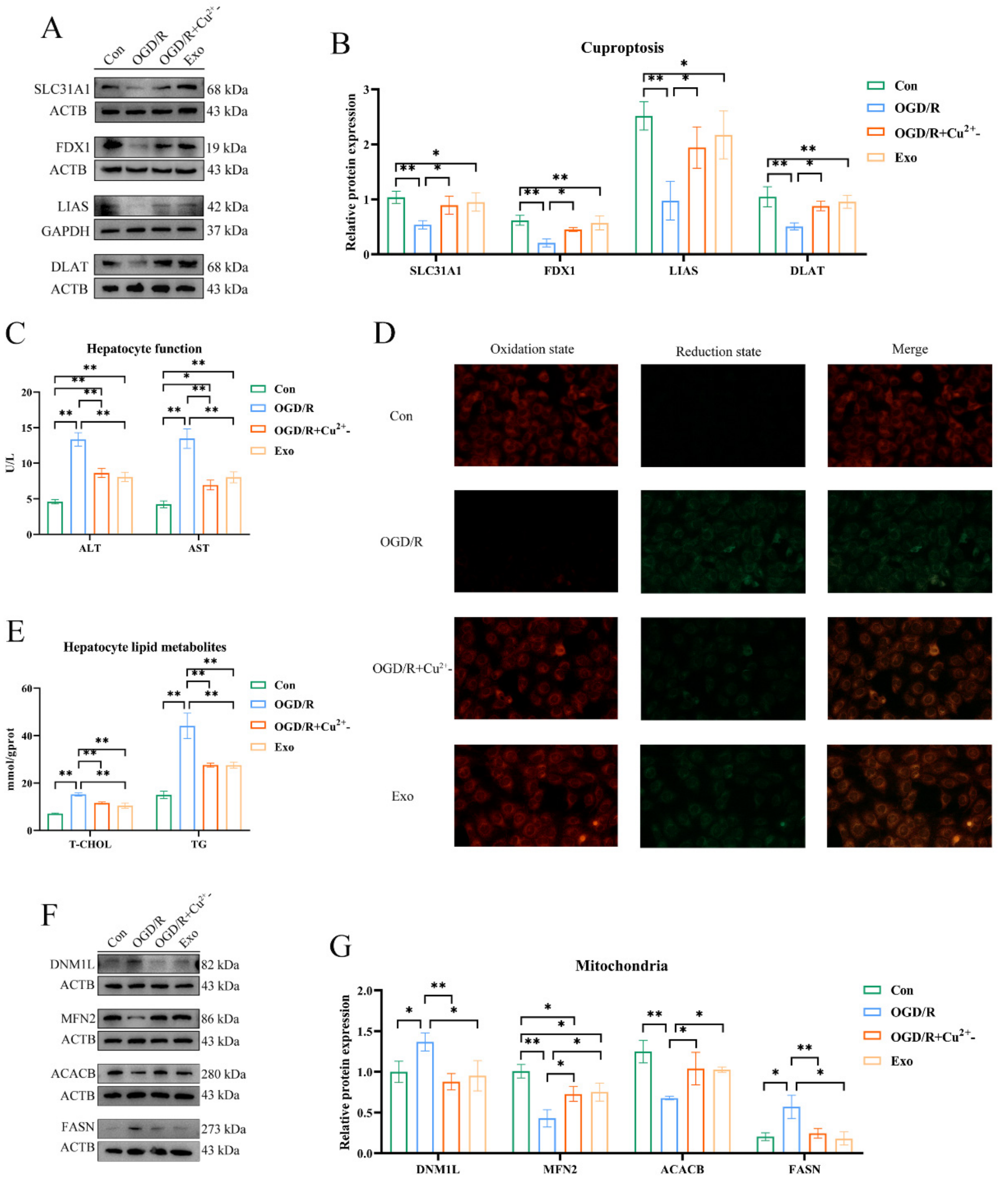

Figure S2. ADSCs-Exo and cuproptosis inhibition alleviate OGD/R-induced hepatocyte injury, mitochondrial dysfunction, and ferroptosis-associated alterations.

Note: (A) Representative western blot images of cuproptosis-related proteins. (B) Quantification of CTR1, FDX1, LIAS, and DLAT protein expression. (C) ALT and AST levels. (D) Cellular redox status assessed by fluorescence staining. (E) T-CHOL and TG levels. (F) Representative western blot images of mitochondrial dynamics- and lipid metabolism-related proteins. (G) Quantification of Drp1, MFN2, ACACB, and FASN protein expression. The data were expressed as mean ± SD. * 0.01 < P < 0.05, ** P < 0.01, n = 3 independent experiments.

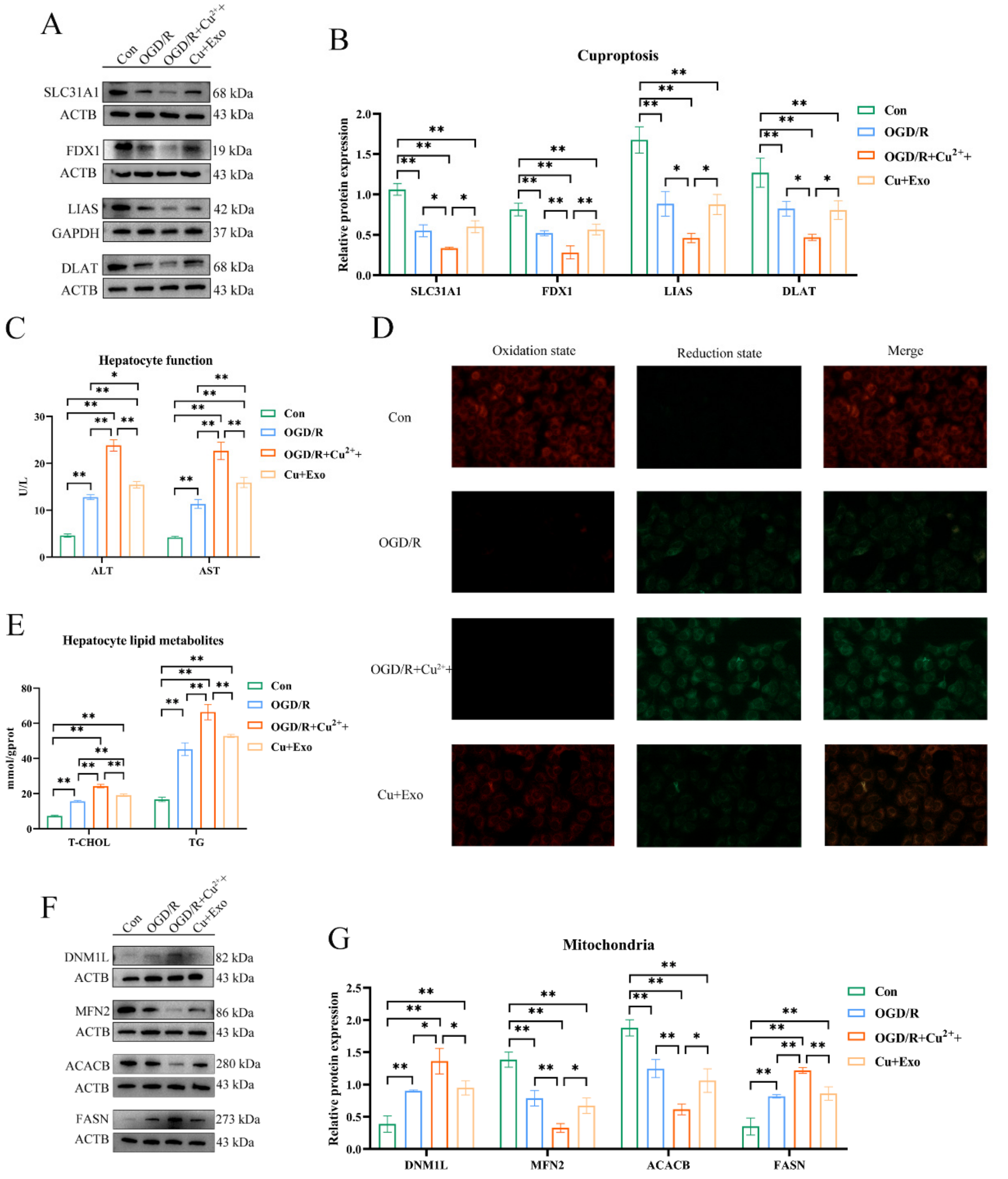

Figure S3. Enhanced cuproptosis aggravates OGD/R-induced hepatocyte injury, mitochondrial dysfunction, and ferroptosis-associated alterations, which are ameliorated by ADSCs-Exo.

Note: (A) Representative western blot images of cuproptosis-related proteins. (B) Quantification of CTR1, FDX1, LIAS, and DLAT protein expression. (C) ALT and AST levels. (D) Cellular redox status assessed by fluorescence staining. (E) T-CHOL and TG levels. (F) Representative western blot images of mitochondrial dynamics- and lipid metabolism-related proteins. (G) Quantification of Drp1, MFN2, ACACB, and FASN protein expression. The data were expressed as mean ± SD. * $0.01 < P < 0.05$, ** $P < 0.01$, n = 3 independent experiments.

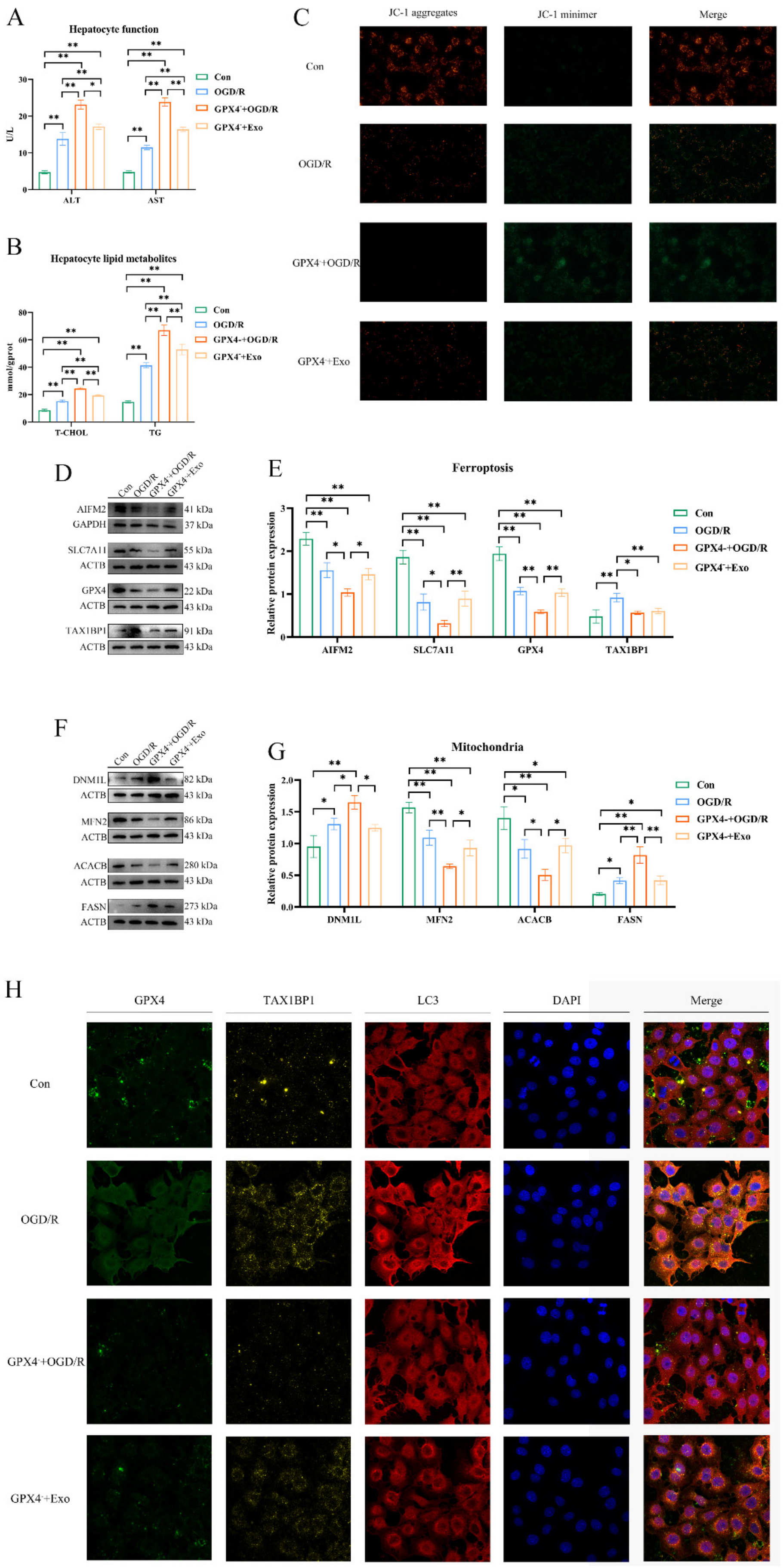

A
Hepatocyte function
U/L
ALT
AST
Con
OGD/R
GPX4-+OGD/R
GPX4-+Exo
B
Hepatocyte lipid metabolites
mmol/gprot
T-CHOL
TG
C
JC-1 aggregates
JC-1 minimer
Merge
Con
OGD/R
GPX4-+OGD/R
GPX4-+Exo
D
AIFM2
GAPDH
SLC7A11
ACTB
GPX4
TAX1BP1
41 kDa
37 kDa
55 kDa
43 kDa
22 kDa
91 kDa
E
Ferroptosis
Relative protein expression
AIFM2
SLC7A11
GPX4
TAX1BP1
F
DNM1L
MFN2
ACACB
FASN
82 kDa
86 kDa
280 kDa
273 kDa
G
Mitochondria
H
GPX4
TAX1BP1
LC3
DAPI
Merge

Figure S4. Ferroptosis enhancement aggravates OGD/R-induced hepatocyte injury, mitochondrial dysfunction, and cuproptosis-associated alterations, which are ameliorated by ADSCs-Exo.

Note: (A) ALT and AST levels. (B) T-CHOL and TG levels. (C) Mitochondrial membrane potential assessed by JC-1 staining. (D) Representative western blot images of ferroptosis-related proteins. (E) Quantification of AIFM2, SLC7A11, and GPX4 protein expression. (F) Representative western blot images of mitochondrial dynamics- and lipid metabolism-related proteins. (G) Quantification of Drp1, MFN2, ACACB, and FASN protein expression. The data were expressed as mean ± SD. * $0.01 < P < 0.05$, ** $P < 0.01$, n = 3 independent experiments.

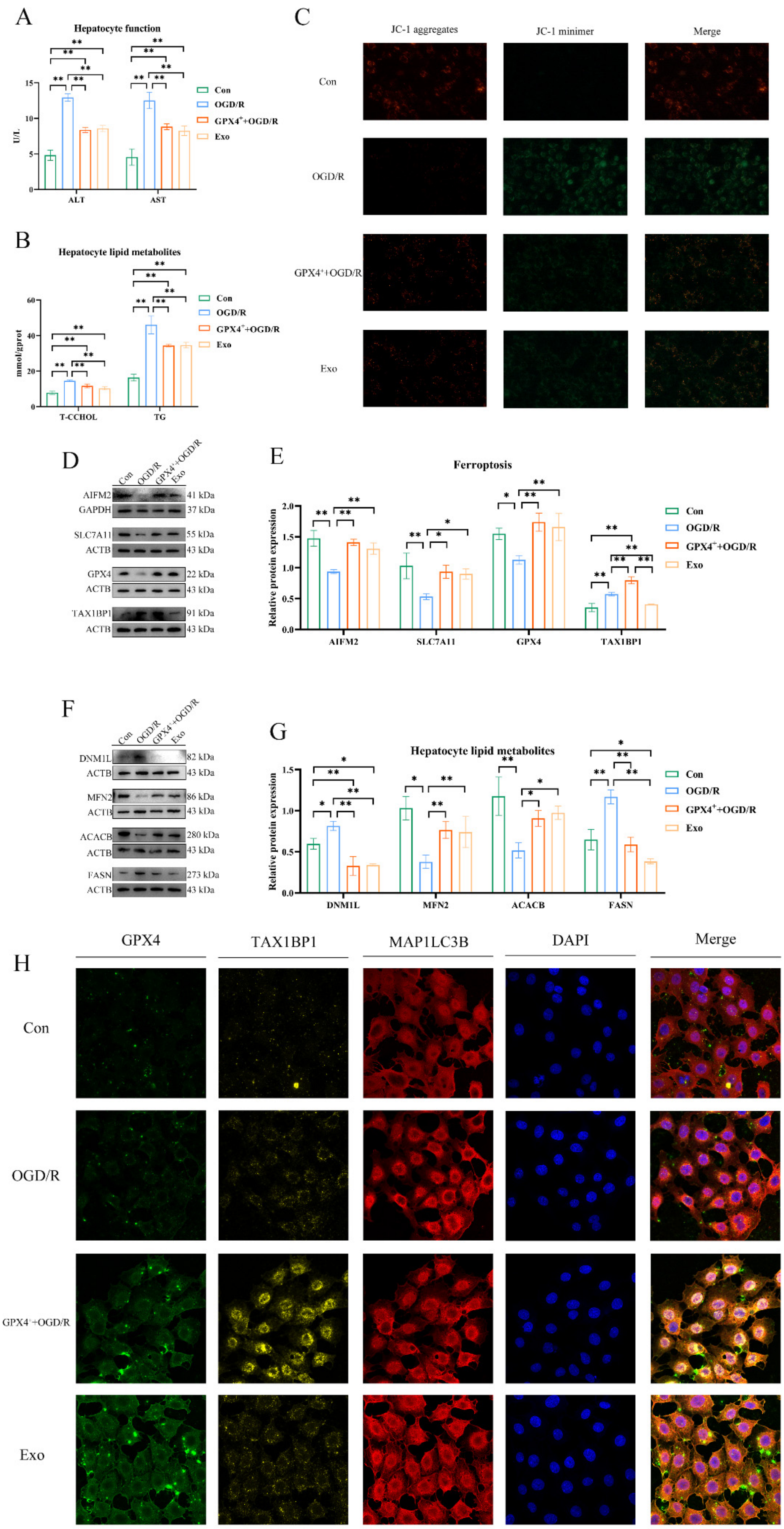
A
Hepatocyte function
U/L
ALT
AST
Con
OGD/R
GPX4+OGD/R
Exo
B
Hepatocyte lipid metabolites
mmol/gprot
T-CCHOL
TG
C
JC-1 aggregates
JC-1 minimer
Merge
D
AIFM2
GAPDH
SLC7A11
ACTB
GPX4
TAX1BP1
41 kDa
37 kDa
55 kDa
43 kDa
22 kDa
91 kDa
E
Ferroptosis
Relative protein expression
F
DNM1L
MFN2
ACACB
FASN
82 kDa
86 kDa
280 kDa
273 kDa
G
Hepatocyte lipid metabolites
H
MAP1LC3B
DAPI

Figure S5. ADSCs-Exo and ferroptosis suppression alleviate OGD/R-induced hepatocyte injury, mitochondrial dysfunction, and cuproptosis-associated alterations.

Note: (A) ALT and AST levels. (B) T-CHOL and TG levels. (C) Mitochondrial membrane potential assessed by JC-1 staining. (D) Representative western blot images of ferroptosis-related proteins. (E) Quantification of AIFM2, SLC7A11, and GPX4 protein expression. (F) Representative western blot images of mitochondrial dynamics- and lipid metabolism-related proteins. (G) Quantification of Drp1, MFN2, ACACB, and FASN protein expression. The data were expressed as mean ± SD. * 0.01 < P < 0.05, ** P < 0.01, n = 3 independent experiments.

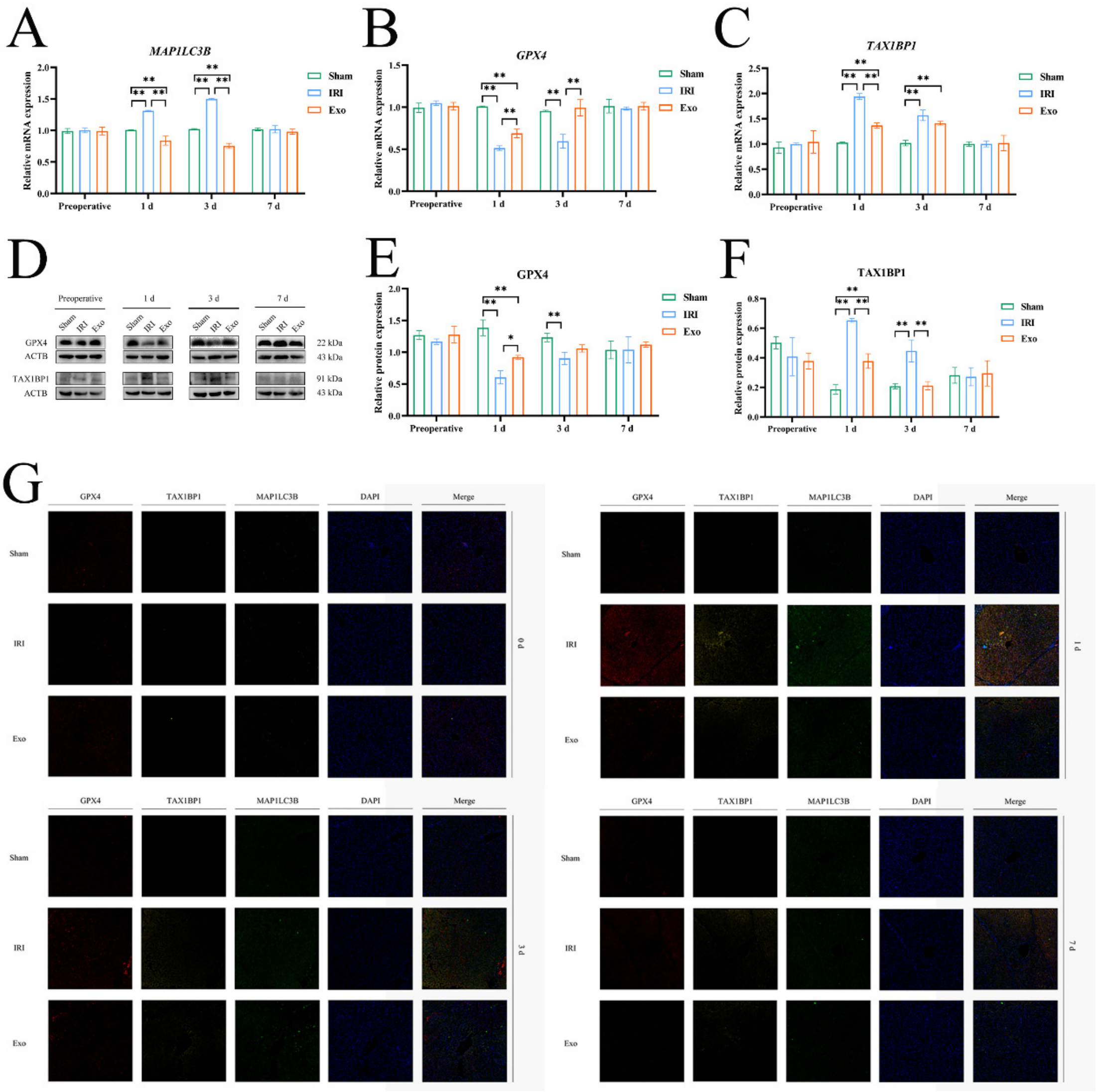

Figure S6 ADSCs-Exo restore GPX4 expression and ameliorate TAX1BP1/MAP1LC3B-associated autophagic alterations following hepatic ischemia-reperfusion injury in miniature pigs.

Note: (A–C) Relative mRNA expression of *GPX4*, *TAX1BP1*, and *MAP1LC3B*, respectively. (D) Representative western blot images of GPX4 and TAX1BP1. (E, F) Quantification of GPX4 and TAX1BP1 protein expression, respectively. (G) Representative immunofluorescence images of GPX4,

TAX1BP1, and MAP1LC3B in liver tissues. The data were expressed as mean ± SD. * $0.01 < P < 0.05$, ** $P < 0.01$, $n = 3$ independent animals per group, randomly selected from the six animals included in each experimental group.
