# Supplementary material for: ADSCs-Exo Attenuate HIRI in Miniature Pigs: Pharmacological Evidence for a Potential Association Between Copper-Associated Alterations and Ferroptosis-Related Responses
Source: Antioxidants (Basel). 2026 Oct 16;15(10):1335. doi: 10.3390/antiox15101335 (PMC13701778; doi:10.3390/antiox15101335)
Supplement: Supplementary file 1 [file antioxidants-15-01335-s001.zip › Supplementary table.pdf]

Table S1. ACTB Ct values

		0 d	1 d	3 d	7 d
ACTB of CSLC31A1	Sham	23.24	23.27	23.34	23.36
		23.06	23.19	23.18	23.33
		23.36	23.35	23.30	23.19
	IRI	23.21	23.03	23.08	23.24
		23.34	23.10	23.19	23.08
		23.09	23.24	23.24	23.13
	Exo	23.28	23.31	23.22	23.27
		23.22	23.32	23.37	23.14
		23.04	23.39	23.47	23.40
ACTB of FDX1	Sham	23.35	23.23	23.11	23.25
		23.20	23.09	23.19	23.14
		23.14	23.18	23.41	23.44
	IRI	23.25	23.22	23.16	23.16
		23.01	23.36	23.29	23.09
		23.29	23.27	23.07	23.39
	Exo	23.28	23.02	23.33	23.31
		23.15	23.15	23.54	23.45
		23.35	23.38	23.39	23.16
ACTB of LIAS	Sham	23.12	23.07	23.11	23.19
		23.19	23.18	23.37	23.02
		23.26	23.26	23.08	23.30
	IRI	23.07	23.15	23.32	23.05
		23.35	23.27	23.06	23.25
		23.22	23.06	23.23	23.19
	Exo	23.09	23.35	23.21	23.17
		23.35	23.04	23.25	23.24
		23.24	23.21	23.08	23.31
ACTB of DLAT	Sham	23.25	23.33	23.21	23.04
		23.03	23.16	23.18	23.15
		23.13	23.27	23.22	23.28
	IRI	23.06	23.25	23.05	23.26
		23.24	23.13	23.16	23.09
		23.13	23.22	23.32	23.21
	Exo	23.36	23.08	23.38	23.18
		23.27	23.15	23.24	23.22
		23.13	23.24	23.28	23.27
ACTB of	Sham	23.55	23.12	23.18	23.11
		23.14	23.19	23.31	23.18
		23.34	23.26	23.16	23.38
	IRI	23.25	23.17	23.41	23.17
		23.31	23.35	23.12	23.12

DNM1L		23.13	23.22	23.35	23.33
	Exo	23.28<br>23.15<br>23.35	23.09<br>23.35<br>23.19	23.27<br>23.22<br>23.13	23.30<br>23.09<br>23.19
ACTB of MFN2	Sham	23.15<br>23.43<br>23.34	23.14<br>23.25<br>23.38	23.36<br>23.18<br>23.22	23.13<br>23.09<br>23.32
	IRI	23.34<br>23.42<br>23.32	23.33<br>23.04<br>23.15	23.32<br>23.18<br>23.19	23.04<br>23.40<br>23.25
	Exo	23.42<br>23.27<br>23.21	23.37<br>23.05<br>23.19	23.25<br>23.39<br>23.28	23.26<br>23.37<br>23.45
ACTB of ACACB	Sham	23.77<br>23.40<br>23.54	23.23<br>23.01<br>23.19	23.09<br>23.22<br>23.45	23.41<br>23.12<br>23.06
	IRI	23.05<br>23.35<br>23.29	23.09<br>23.06<br>23.08	23.42<br>23.34<br>23.52	23.27<br>23.06<br>23.38
	Exo	23.50<br>23.39<br>23.41	23.09<br>23.23<br>23.67	23.47<br>23.43<br>23.30	23.45<br>23.15<br>23.24
ACTB of FASN	Sham	23.21<br>23.09<br>23.25	23.09<br>23.10<br>23.21	23.21<br>23.37<br>23.45	23.30<br>23.12<br>23.06
	IRI	23.38<br>23.22<br>23.18	23.40<br>23.15<br>23.07	23.14<br>23.36<br>23.32	23.20<br>23.03<br>23.06
	Exo	23.41<br>23.31<br>23.20	23.37<br>23.23<br>23.36	23.47<br>23.21<br>23.35	23.39<br>23.09<br>23.25
ACTB of AIFM2	Sham	23.32<br>23.39<br>23.27	23.45<br>23.36<br>23.56	23.11<br>23.18<br>23.07	23.01<br>23.26<br>23.17
	IRI	23.48<br>23.35<br>23.17	23.27<br>23.10<br>23.03	23.36<br>23.25<br>23.21	23.43<br>23.27<br>23.35
	Exo	23.10<br>23.21<br>23.37	23.02<br>23.23<br>23.12	23.09<br>23.39<br>23.45	23.32<br>23.19<br>23.12
	Sham	23.16<br>23.26<br>23.30	23.16<br>23.31<br>23.40	23.39<br>23.27<br>23.23	23.31<br>23.39<br>23.23
		23.40	23.31	23.16	23.34

ACTB of TFRC	IRI	23.26<br>23.13	23.19<br>23.12	23.10<br>23.18	23.37<br>23.39
	Exo	23.07<br>23.06<br>23.09	23.19<br>23.41<br>23.19	23.48<br>23.46<br>23.43	23.16<br>23.05<br>23.04
ACTB of GPX4	Sham	23.23<br>23.55<br>23.46	23.37<br>23.25<br>23.15	23.60<br>23.75<br>23.77	23.25<br>23.12<br>23.03
	IRI	23.03<br>23.19<br>23.22	23.64<br>23.53<br>23.61	23.48<br>23.45<br>23.46	23.36<br>23.18<br>23.05
	Exo	23.36<br>23.22<br>23.31	23.40<br>23.42<br>23.60	23.65<br>23.64<br>23.74	23.27<br>23.38<br>23.47
ACTB of SLC7A11	Sham	23.37<br>23.24<br>23.16	23.29<br>23.21<br>23.16	23.46<br>23.18<br>23.22	23.19<br>23.34<br>23.12
	IRI	23.23<br>23.17<br>23.26	23.08<br>23.40<br>23.27	23.19<br>23.15<br>23.09	23.19<br>23.12<br>23.26
	Exo	23.19<br>23.24<br>23.31	23.24<br>23.06<br>23.18	23.27<br>23.39<br>23.24	23.04<br>23.23<br>23.09
ACTB of MAP1LC3B	Sham	23.20<br>23.40<br>23.17	23.23<br>23.19<br>23.2	23.09<br>23.05<br>23.04	23.41<br>23.21<br>23.26
	IRI	23.53<br>23.59<br>23..69	23.30<br>23.19<br>23.14	23.42<br>23.53<br>23.34	23.27<br>23.31<br>23.34
	Exo	23.50<br>23.39<br>23.41	23.09<br>23.23<br>23.19	23.47<br>23.43<br>23.30	23.26<br>23.22<br>23.47
ACTB of TAX1BP1	Sham	23.58<br>23.34<br>23.27	23.45<br>23.36<br>23.56	23.15<br>23.19<br>23.25	23.43<br>23.47<br>23.36
	IRI	23.35<br>23.46<br>23.53	23.27<br>23.10<br>23.03	23.36<br>23.25<br>23.21	23.43<br>23.27<br>23.35
	Exo	23.10<br>23.21<br>23.37	23.02<br>23.23<br>23.13	23.24<br>23.39<br>23.38	23.32<br>23.20<br>23.26
